# Supplementary material for: KLF4-PFKFB3-driven glycolysis is essential for phenotypic switching of vascular smooth muscle cells
Source: Commun Biol. 2022 Dec 5;5:1332. doi: 10.1038/s42003-022-04302-y (PMC9722670; doi:10.1038/s42003-022-04302-y)
Supplement: Supplementary file 2 — Supplementary Information [file 42003_2022_4302_MOESM2_ESM.pdf]

Supplementary Information

KLF4-PFKFB3-driven glycolysis is essential for phenotypic switching of vascular smooth muscle cells

Xinhua Zhang, Bin Zheng, Lingdan Zhao, Jiayi Shen, Zhan Yang, Yu Zhang, Ruirui Fan, Manli Zhang, Dong Ma, Lemin Zheng, Mingming Zhao, Huirong Liu, Jinkun Wen

Supplementary Tables 1-4
Supplementary Figures 1-20

Supplementary Tables

Supplementary Table 1 Primers for circ-pcDNA3.1 vector construct.

name	Sequences 5' to 3'	Amplicon (bp)
5' Alu sequences	CGGGCGCGGTGCCTCACGCCTGTAATCCCAGCACTTTGGGAGCCTGGGGC	N/A
3' Alu sequences	GCCCCAGGCTCCCAAAGTGCTGGGATTACAGGCGTGAGGCACCGCGCCCG	
5' donor splice sequences	ACTCTGACAATTCCCTTTCTTTCCCTCAG	
3' acceptor splice sequences	GTGAGTATCAACTGCAGACGTTTCGTGCGGC	

Supplementary Table 2 Primers for circRNA expressing plasmid constructs.

circRNA	Primer name	Primer sequences 5' to 3'	Amplicon (bp)
circZFAT (#1)	pcDNA-ZFAT-inf-F:	CATTCCCTTTCTTTCCCTCAGCAGTGCTGTGTGAACTTTAAGATGTGCC	2027
	pcDNA-ZFAT-inf-R:	CGTCTGCAGTTGATACTCACGTAACGAGTCTGACCTTGAACTAGAAAAGAAGT	
circCTDP1 (#2)	pcDNA-CTDP1-inf-F:	CATTCCCTTTCTTTCCCTCAGAGCGGTTCTGGTGAGGTTGGAAGG	458
	pcDNA-CTDP1-inf-R:	CGTCTGCAGTTGATACTCACCTGCGATGGTGTGTGCGTACAGC	
circCUX1 (#3)	pcDNA-CUX1-inf-F:	CATTCCCTTTCTTTCCCTCAGGGCCGACGAGATTGAAATGATCATGACGG	343
	pcDNA-CUX1-inf-R:	CGTCTGCAGTTGATACTCACTTTGAGTGTGCTGTTTTTGGCGCTCAGC	
circRPS6KA1 (#4)	pcDNA-RPS6KA1-inf-F:	CATTCCCTTTCTTTCCCTCAGCACCACCTCCGTACCTTGTTGGGG	220
	pcDNA-RPS6KA1-inf-R:	CGTCTGCAGTTGATACTCACTGCTCTGTGTCTCCACGGTCCACACC	
circTM4SF-TCTEX1D2 (#5)	pcDNA-TM4SF-TCTEX1D2-inf-F:	CATTCCCTTTCTTTCCCTCAGCCACTCCTTGTAGAAAGGATTCAAGACAGCC	424
	pcDNA-TM4SF-TCTEX1D2-inf-R:	CGTCTGCAGTTGATACTCACGTGCTTACTGCTCTGTTGTCAGGTGG	
circTSNARE1 (#6)	pcDNA-TSNARE1-inf-F:	CATTCCCTTTCTTTCCCTCAGCTCCATCTGCAGGATGGCCTCCTC	397

	pcDNA-TSNARE1-inf-R:	CGTCTGCAGTTGATACTCACGCACACGGCACAGCAGGAGACC	
circDDX42 (#7)	pcDNA-DDX42-inf-F:	CATTCCCTTTCTTTCCCTCAGATTGACTATCCACCATTTGAAAAAAACTTTTACAATG	225
	pcDNA-DDX42-inf-R:	CGTCTGCAGTTGATACTCACTGGCACTGTATTGGAGTGGGCTGTGTG	
circTNRC6B (#8)	pcDNA-TNRC6B-inf-F:	CATTCCCTTTCTTTCCCTCAGTCTGGAGCAAAAGCACACCACCTG	335
	pcDNA-TNRC6B-inf-R:	CGTCTGCAGTTGATACTCACCTTCATTTGTTTCTTTCCTCCTTGTCCC	
circZBTB46 (#9)	pcDNA-ZBTB46-inf-F:	CATTCCCTTTCTTTCCCTCAGGGGACAGCGAGTGGGCCCCTC	1255
	pcDNA-ZBTB46-inf-R:	CGTCTGCAGTTGATACTCACAGTCTGTAGAAGAGGCGACACCAGGGCTTCC	
circADAMTS17 (#10)	pcDNA-ADAMTS17-inf-F:	CATTCCCTTTCTTTCCCTCAGCTCCATGTTTCTGCAGAAGGTGGCATTC	292
	pcDNA-ADAMTS17-inf-R:	CGTCTGCAGTTGATACTCACCTTGGGCATGAACCACGACGATGACC	
circTMEM209 (#11)	pcDNA-TMEM209-inf-F:	CATTCCCTTTCTTTCCCTCAGCTCCTATCTGTAGCTCTGGACAACCCATTCG	547
	pcDNA-TMEM209-inf-R:	CGTCTGCAGTTGATACTCACTTGGCGAGCTTTAGCCCCTCTCCTCC	
circCNDP2 (#12)	pcDNA-CNDP2-inf-F:	CATTCCCTTTCTTTCCCTCAGAACCTTCGAGATCTGCGGTCTGGGGTCTGG	241
	pcDNA-CNDP2-inf-R:	CGTCTGCAGTTGATACTCACCTTTTGTTTTCCGATATCCACCAGTTCC	
circTEX2 (#13)	pcDNA-TEX2-inf-F:	CATTCCCTTTCTTTCCCTCAGCTTGGTGATCAACGTAAGGCTTGAAGG	495
	pcDNA-TEX2-inf-R:	CGTCTGCAGTTGATACTCACGGCTTTTGCCTGCACACAGCAGAC	
circFAM53B-1 (#14)	pcDNA-FAM53B-1-inf-F:	CATTCCCTTTCTTTCCCTCAGCCATAATTCCACAAGAGAAAAGTGTAGGTCC	307
	pcDNA-FAM53B-1-inf-R:	CGTCTGCAGTTGATACTCACGGAGTTGACCACATTTGGCCATTTCCC	
circFAM53B-2 (#15)	pcDNA-FAM53B-2-inf-F:	CATTCCCTTTCTTTCCCTCAGCAGTTCACGGCTGAAGGTCCCAC	252
	pcDNA-FAM53B-2-inf-R:	CGTCTGCAGTTGATACTCACGGAGTTGACCACATTTGGCCATTTCCC	

Supplementary Table 3 Primers for qRT-PCR.

name	Sequences 5' to 3'	Amplicon (bp)
CD123	F: ACGCCGACTATTCTATGCC<br>R: GGAAATTGCTCCAAACTGG	59
CXCR4	F: ATAAAATCTTCCTGCCCACC<br>R: TACTTGTCCGTCATGCTTCTC	118
LILRB2	F: ATGACAGGAGCCTACCCAAAA<br>R: ACAGAATGAAGCCGCCAAA	118
LILRB3	F: GTCAGCGTCACAGCAAACA<br>R: GTCCTCAGACTGTGTGTCCTTC	176
HMHA1	F: CGGGTCAATGGGGTAAAGA<br>R: TGGTAGAGGCGGAAGGAGAT	164
CD52	F: CGCTTCCTCTTCCTCCTACT<br>R: AATGCCTCCGCTTATGTTG	129
LAG3	F: TTGGCTTTCACCTTTGGAG<br>R: GGCAGAAAATCGTCTTGGTC	50
CD83	F: TCAAGTTATTGGAGGGTGGTG<br>R: GTAGTGTTTCGGATCTTCAGGG	145
CD24	F: GAACCTGGTCCTAAGCCTAAA<br>R: AAAAGGTTTATGTGTGTCGAGG	79
TNFSF9	F: CCAAAATGTTCTGCTGATCG<br>R: CAAGTGAAACGGAGCCTGA	200
LY6D	F: CTCCAGCAACTGCAAGCAT<br>R: CCGCACAGTCCTTCTTCAC	110
TNFAIP2	F: TACGCTGGCCGAGATCATTC<br>R: TCCCCTTGATGGCCAGGATA	125
ZBTB46	F: AGCAGGTTTCACCGTCTCAG<br>R: GCTGTCATCTTCCACCTGCT	179
PFKFB3 primer 1	F: CAGAGCCGAGTGCAGAAGAT<br>R: GAACACTTTTGTGGGGACGC	189
PFKFB3 primer 2	F: AAACTGACGCCTGTCGCTTA<br>R: TCCGGGAGCCTTTCATGTTT	280
eEF1A2	F: TGTGGGGACAGCAAGTCTGA<br>R: ATGATGACCTGGGAGGTGAACT	62
KLF4	F: GCTGTGGATGGAAATTCGCC<br>R: GGTGGTCCGACCTGGAAAAT	117
CTDP1	F: AATAAAGGCATCTTTCACTTCCAG<br>R: TACAGCTTGGCGATCTTCTCC	104
ZFAT	F: TGTCATTATTCTTCCATCACCA<br>R: GGAGTTGAGTAGTCACAGCCA	107

β-actin	F: GTGGCCGAGGACTTTGATTG<br>R: CCTGTAACAACGCATCTCATATT	73
circCCNI	F: GCCACACCATTGGATTTTCTTC<br>R: GCATTTTCCGCACATTCACTT	173
circDDX42	F: ACACAGCCCACTCCAATACAG<br>R: GAGACCCGAAGATTGAGCTTAT	137
circZFAT	F: CCACCGATGGCTGTGACTACT<br>R: TTCCCGATCATCTTCCTTACACT	126
circTMEM209	F: CACAGATGAGACGAATGGGTTG<br>R: CAGTGGTAGGGTACGGAGAAGG	88
circCUX1	F: TGGTGGAGGACGTGCAGAG<br>R: GGAGTGATTGGCCGATGAGA	228
circZBTB46	F: GATGACGGCTCCCTGCTGTTC<br>R: GCCCTGGTGTCGCCTCTTCTA	82
circADAMTS17	F: CCGGGCATGCACTACAGT<br>R: CTTTCACCCACTCTCCTGACA	155
circCDK14	F: GCACTGACAAGCAGCACATTC<br>R: TACAGGGTAAAGCGTTCTGGC	115
circOVOL2	F: GCCACAACCAGGTGAAAAGAC<br>R: TTGAGGTGACGGTTCAGCAT	188
circTEX2	F: CAAGCCTTACGTTGATCACCA<br>R: TGACATGATCTCCTCCACACTG	118
circCTDP1	F: CGAGCTGCACGTCTTCACCT<br>R: CAACCTCACCAGAACCGCTC	72
circPITPNB	F: CCAGATGTGTGCTTGTGATGATAC<br>R: TTGACTGACTGGAATAATGCTGG	82
circTNRC6B	F: CCTCCCACAACTCAGCAAG<br>R: CCCAAGCGGAAGTACCAT	101
circSTMN3	F: AGGAAGCCTACAAGGAGAAGATG<br>R: ACTGGTAGACGGTATTGGGGTG	96
circTDRD12	F: CCGCAGACCAGTACCTGGCA<br>R: ACACCCTTTTATAATAACCCAGA	232
circTSNARE1	F: CGGACATCACTGAAGAGGACCT3'<br>R: CTGCAATGGTCTTGTTGGTCTC3'	103
circRPS6KA1	F: AGATAGGCGGACGCTCTTGG<br>R: GATCACCAGGTTACCCCAACAAG	88
circCNDP2	F: TGCGTGGCCGGAGAAGAGAG<br>R: AAACAGGGTAGTGAGGGCCGC	170
circPREB	F: GCACACCTTACCGCTACCA<br>R: GCAAGGAGGCACTCAAGC	77
circTM4SF19-TCTEX1D2	F: TTCATGTCATCAACAGCCTCC<br>R: TGACAACAGAGCAGTAAGCACC	147
circFAM53B	F: GTGATGGTCCTAAGTGAAAGCC<br>R: GGGAATTGATGTCAGCAGAAAC	165

Supplementary Table 4 The siRNA sequences.

name	Sequences 5' to 3'
si-PFKFB3	GCUGUGAAGCAGUACAGCUCCUACTT<br>GUAGGAGCUGUACUGCUUCACAGCTT
si-PFKFB3#	AAUUUGUCCCCCUUCUUUCGC<br>GAAAGAAGGGGGACAAAUUGC
si-eEF1A2	GCAGGACGUGUACAAGAUUTT<br>AAUCUUGUACACGUCCUGCTT
si-eEF1A2#	CCCAGGUCAUCAUCCUGAATT<br>UUCAGGAUGAUGACCUGGGTT
si-KLF4	UCUUUGUGUAGGUUUUGCCGC<br>GGCAAAACCUACACAAAGAGU
si-KLF4#	ACUCUUUGUGUAGGUUUUGCC<br>CAAAACCUACACAAAGAGUUC
si-circCTDP1	CCAUCGCAGAGCGGUUCUGUU<br>CAGAACCGCUCUGCGAUGGUU
si-circCTDP1#	ACACACCAUCGCAGAGCGGUU<br>CCGCUCUGCGAUGGUGUGUUU
si-circZFAT	UCGUUACCAGUGCUGUGUGUU<br>CACACAGCACUGGUAACGAUU
si-circZFAT#	AGACUCGUUACCAGUGCUGUU<br>CAGCACUGGUAACGAGUCUUU
si-both-C	UGAAAGAUGCCUUUAUUCGAC<br>CGAAUAAAGGCAUCUUUCACU
si-both-C#	AGCUGUUCAGCUUGCUCGGAG<br>CCGAGCAAGCUGAACAGCUGG
si-both-Z	AGAAGAAGCAAAUGAACACCA<br>GUGUUCAUUUGCUUCUUCUGU
si-both-Z#	UGUGAAUACUGCAACAAGGUC<br>CCUUGUUGCAGUAUUCACAAG

Supplementary Figures

Supplementary Figure 1

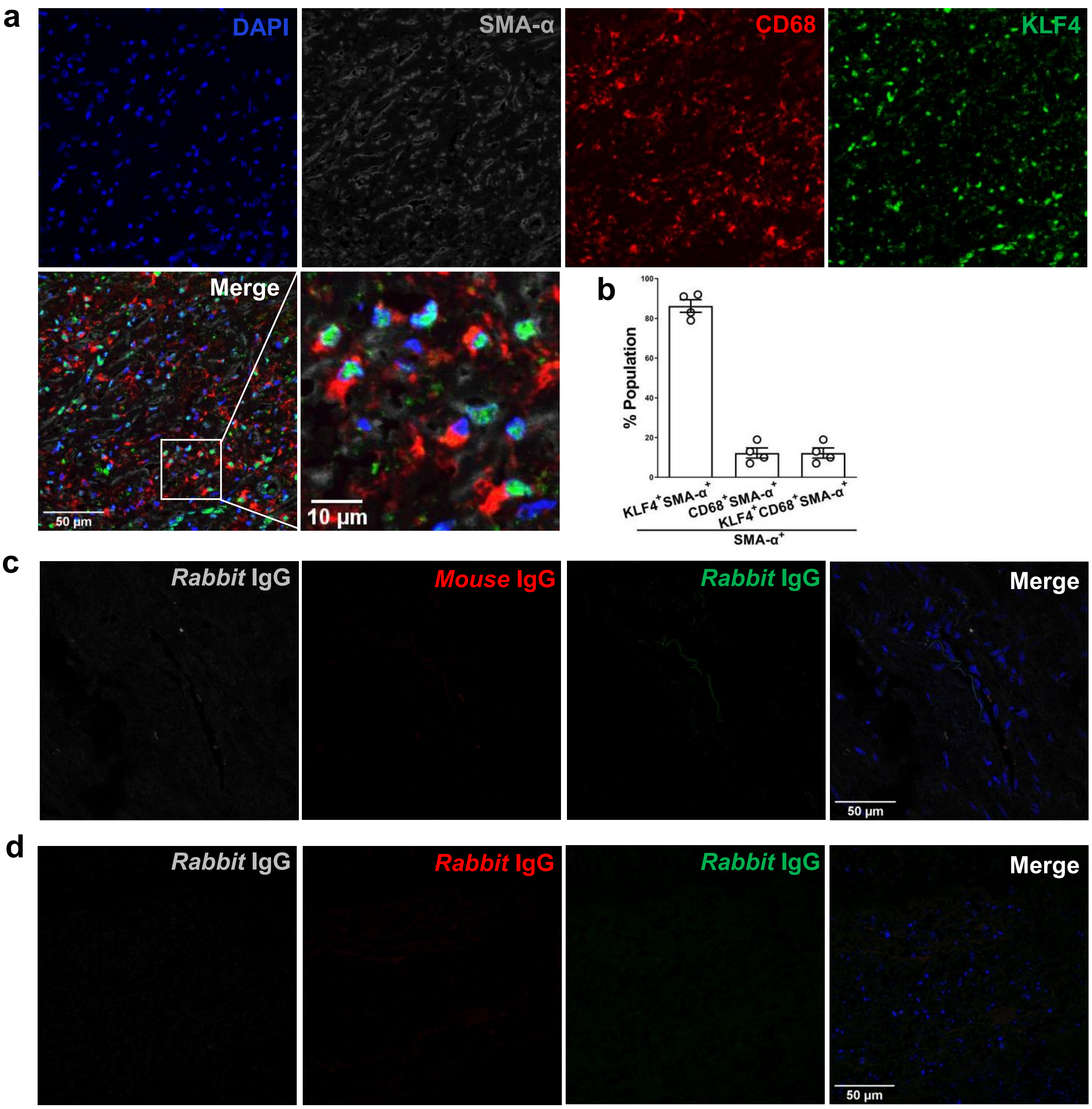

Supplementary Fig. 1 VSMCs within human atherosclerotic lesions express the Mϕ marker CD68. a Representative multiplex IHC staining of the core region of human renal artery atherosclerotic lesions. bars=50 μm. **b** Quantification of the frequency of $KLF4^{+}SMA\text{-}\alpha^{+}$, $CD68^{+}SMA\text{-}\alpha^{+}$, and $KLF4^{+}CD68^{+}SMA\text{-}\alpha^{+}$ cells as a percent of total $SMA\text{-}\alpha^{+}$ cells in the core region of human renal artery atherosclerotic lesions (n=4 independent experiments, error bars show SEM). **c, d** Representative images of quality controls of the multiplex IHC staining. bars=50 μm.

Supplementary Figure 2

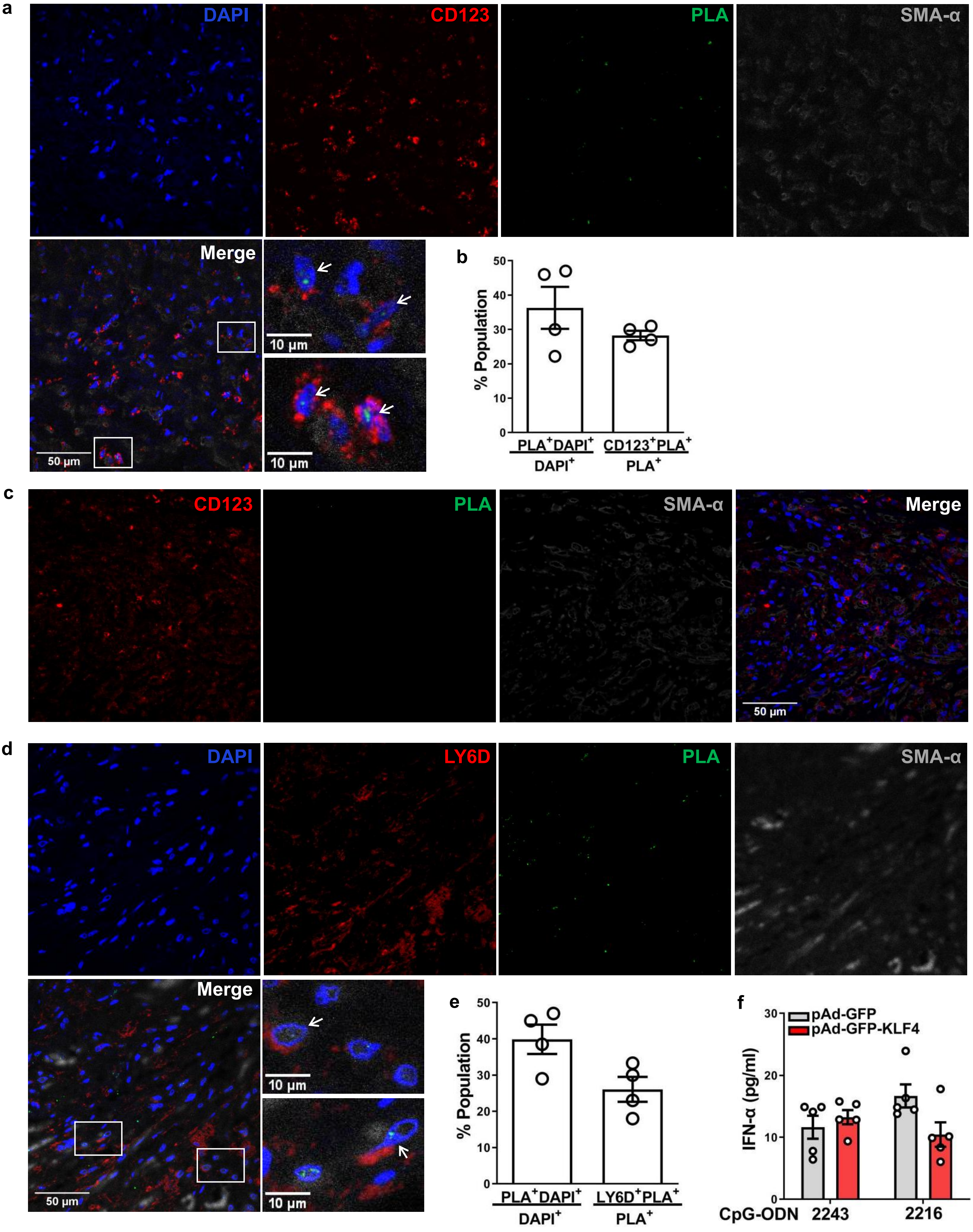

Supplementary Fig. 2 pDC markers CD123 and LY6D are expressed in VSMCs of human atherosclerotic lesions. a-e VSMCs within the core region of human renal artery atherosclerotic lesions were identified based on proximity ligation assay (PLA) detection of the VSMC specific stable epigenetic signature H3K4dime on the *MYH11*. *MYH11* H3K4dime PLA^+ cells exhibit a punctate green dot within the nucleus (**a, d**). Samples were also immuno-stained for CD123 (red) (**a**), LY6D (red) (**d**), as well as SMA-α (grey) and DAPI (blue) (**a, d**). White arrow showed the $CD123^+PLA^+$ or $LY6D^+PLA^+$ cells. ISH-PLA negative control using an empty vector probe showed no PLA amplification (**c**). Quantification (un-corrected) of the frequency of PLA^+DAPI^+ cells as a percent of total $DAPI^+$ cells, $CD123^+PLA^+$ cells as a percent of total PLA^+ cells, as well as $LY6D^+PLA^+$ cells as a percent of total PLA^+ cells in the core region of human renal artery atherosclerotic lesions (n=4 independent experiments, error bars show SEM) (**b, e**). **f** VSMCs were infected with pAd-GFP or pAd-GFP-KLF4 for 24 h, and then treated with the adjuvant CpG ODN (6 μg/ml) for another 24 h. The culture medium was then collected and IFN-α was detected by ELISA. n=5 independent experiments, error bars show SEM. One-way ANOVA with Tukey's multiple comparison tests were performed.

Supplementary Figure 3

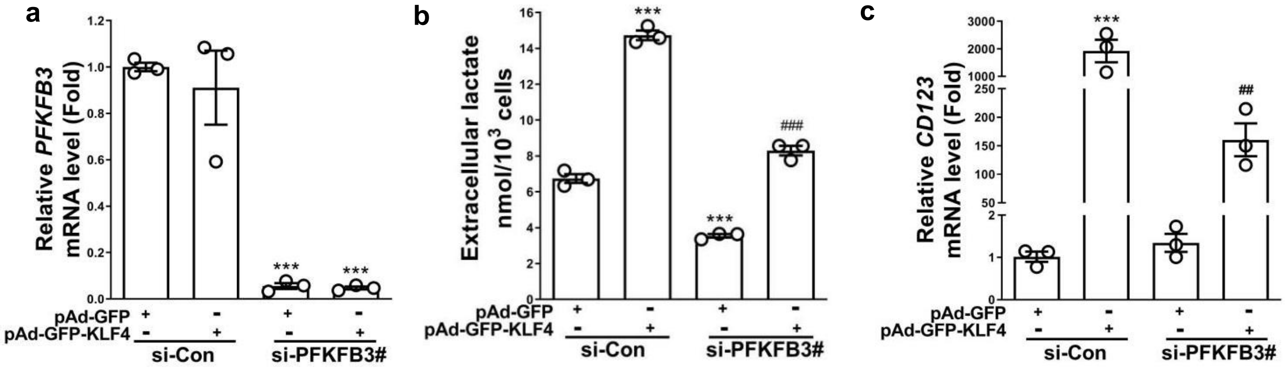

Supplementary Fig. 3 KLF4 enhances glycolysis by upregulating PFKFB3 expression. VSMCs were transfected with the indicated constructs. *PFKFB3* mRNA levels were assessed by qRT-PCR (**a**). Analysis of lactate production levels (**b**). *CD123* mRNA levels were assessed by qRT-PCR (**c**). ****P*<0.005 vs si-Con+pAd-GFP, ##*P*<0.01 and ###*P*<0.005 vs si-Con+pAd-GFP-KLF4 (n=3 independent experiments, error bars show SEM). One-way ANOVA with Tukey's multiple comparison tests were performed.

Supplementary Figure 4

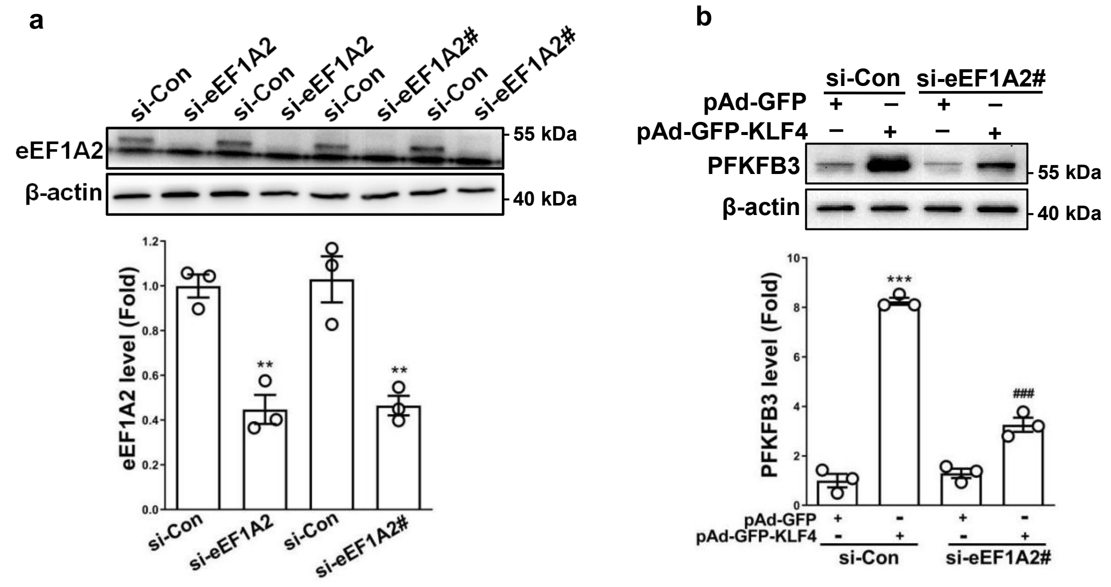

Supplementary Fig. 4 KLF4 upregulates PFKFB3 expression via eEF1A2. a VSMCs were transfected with two siRNAs targeting eEF1A2, si-eEF1A2 and si-eEF1A2#, for 24 h. eEF1A2 protein levels were measured by immunoblotting and quantified by normalizing to β-actin. ***P*<0.01 vs si-Con (n=3 independent experiments, error bars show SEM). Unpaired Student's t-tests were performed. **b** VSMCs were transfected with the indicated constructs. PFKFB3 protein levels were measured by immunoblotting and quantified by normalizing to β-actin. ****P*<0.005 vs si-Con+pAd-GFP, ###*P*<0.005 vs si-Con+pAd-GFP-KLF4 (n=3 independent experiments, error bars show SEM). One-way ANOVA with Tukey's multiple comparison tests were performed.

Supplementary Figure 5

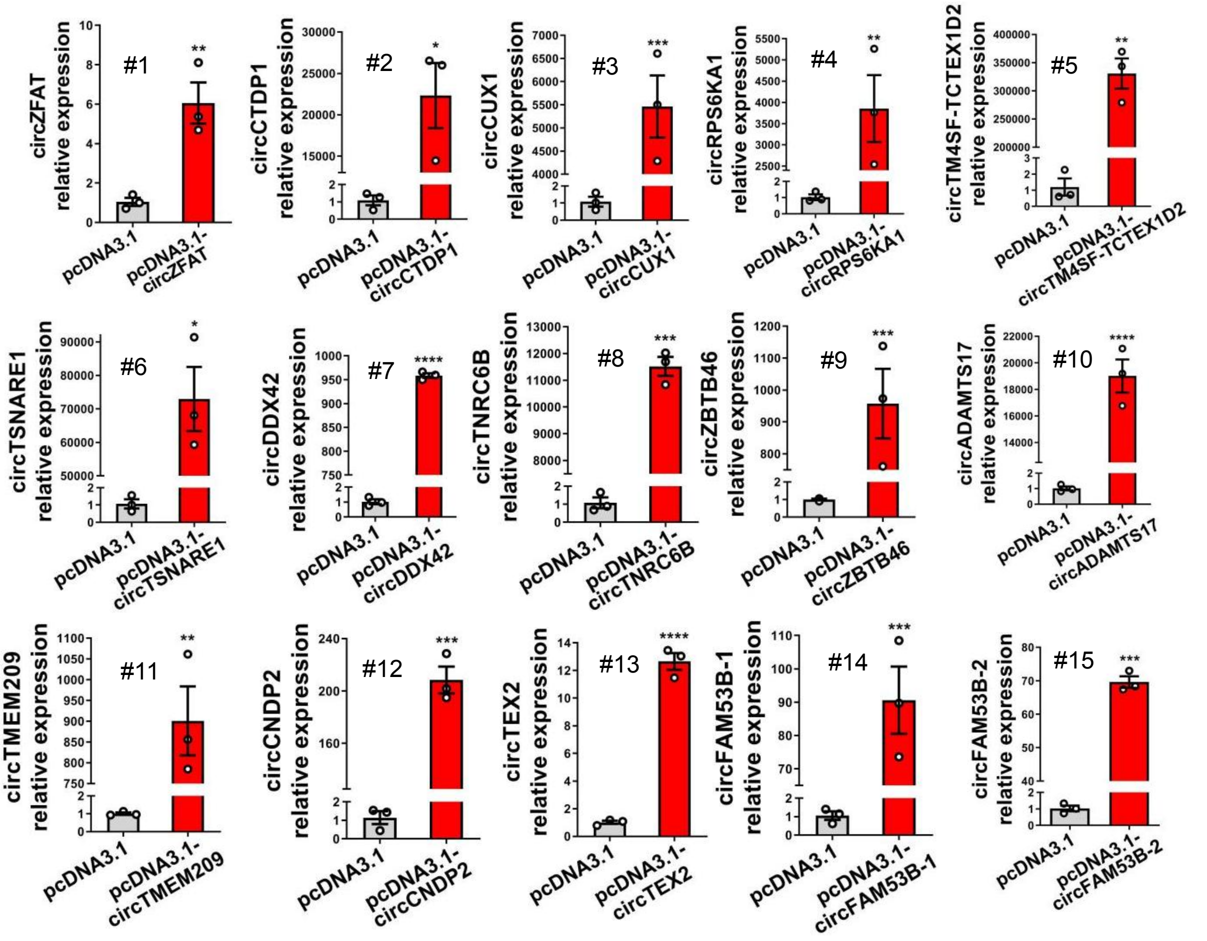

Supplementary Fig. 5 Transfecting VSMCs with circRNA expressing plasmids increases their corresponding circRNA levels. VSMCs were transfected with the indicated circRNA expression plasmids for 48 h. Expression levels for the indicated circRNAs were assessed by qRT-PCR. **P*<0.05, ***P*<0.01, ****P*<0.005 and *****P*<0.001 vs pcDNA3.1 (n=3 independent experiments, error bars show SEM). Unpaired Student's t-tests were performed.

Supplementary Figure 6

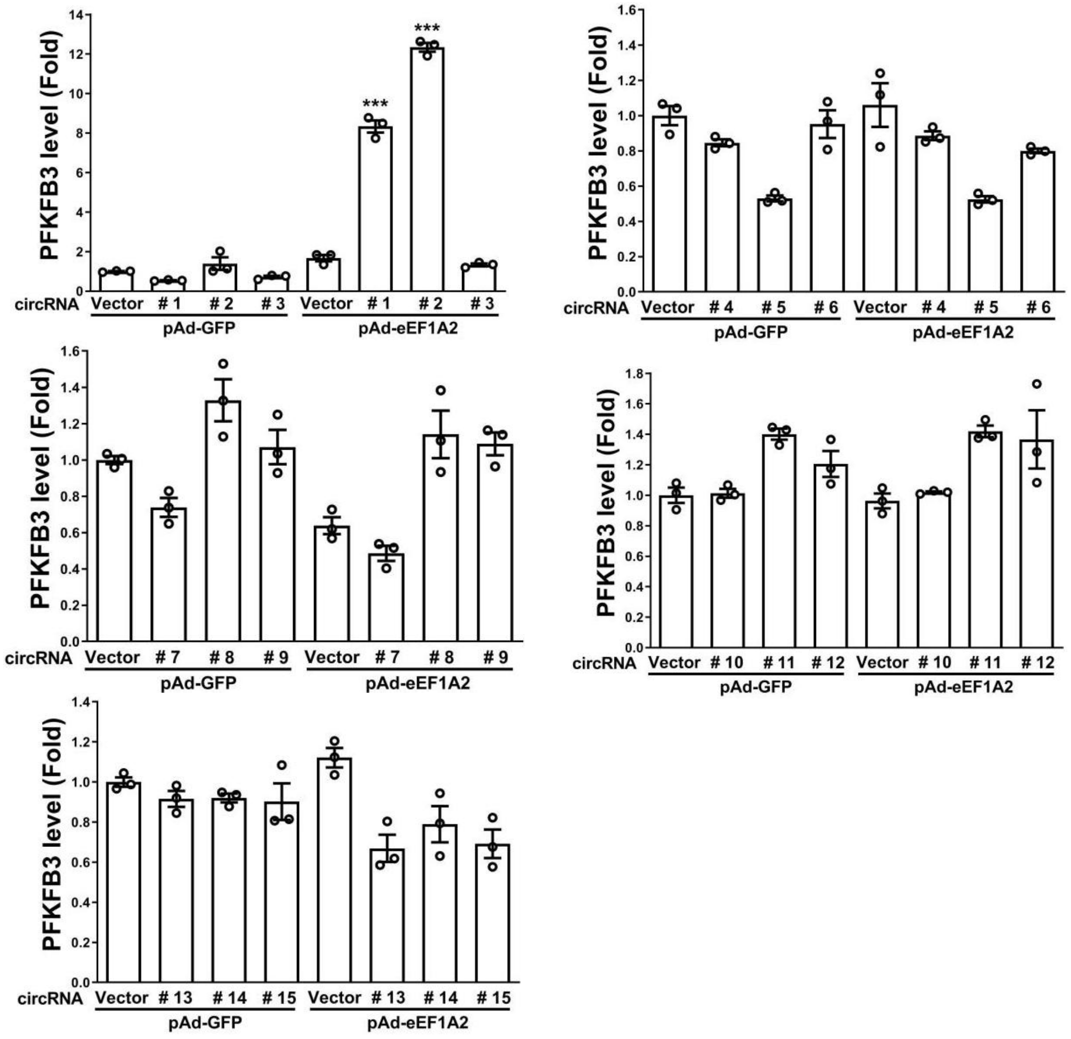

Supplementary Fig. 6 circZFAT (#1) and circCTDP1 (#2) increase PFKFB3 protein levels when co-overexpressed with eEF1A2. VSMCs were transfected with the indicated constructs. PFKFB3 protein levels were measured by immunoblotting and quantified by normalizing to β-actin. ****P*<0.005 vs vector+pAd-eEF1A2 (n=3 independent experiments, error bars show SEM). One-way ANOVA with Tukey's multiple comparison tests were performed.

Supplementary Figure 7

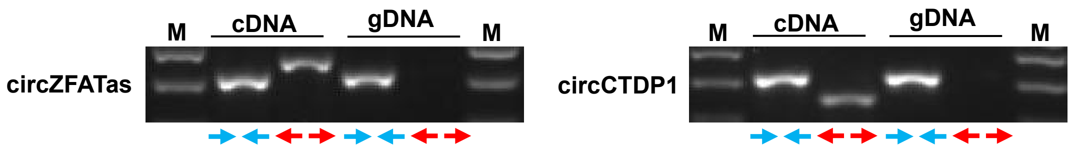

Supplementary Fig. 7 The presence of circZFAT and circCTDP1 was confirmed using divergent primers to amplify circRNAs formed by head-to-tail splicing. Convergent or divergent primers were used to detect the indicated circRNAs via reverse transcription (RT)-PCR in VSMCs. circRNAs were amplified by divergent primers in cDNA but not genomic DNA (gDNA). M=Marker.

Supplementary Figure 8

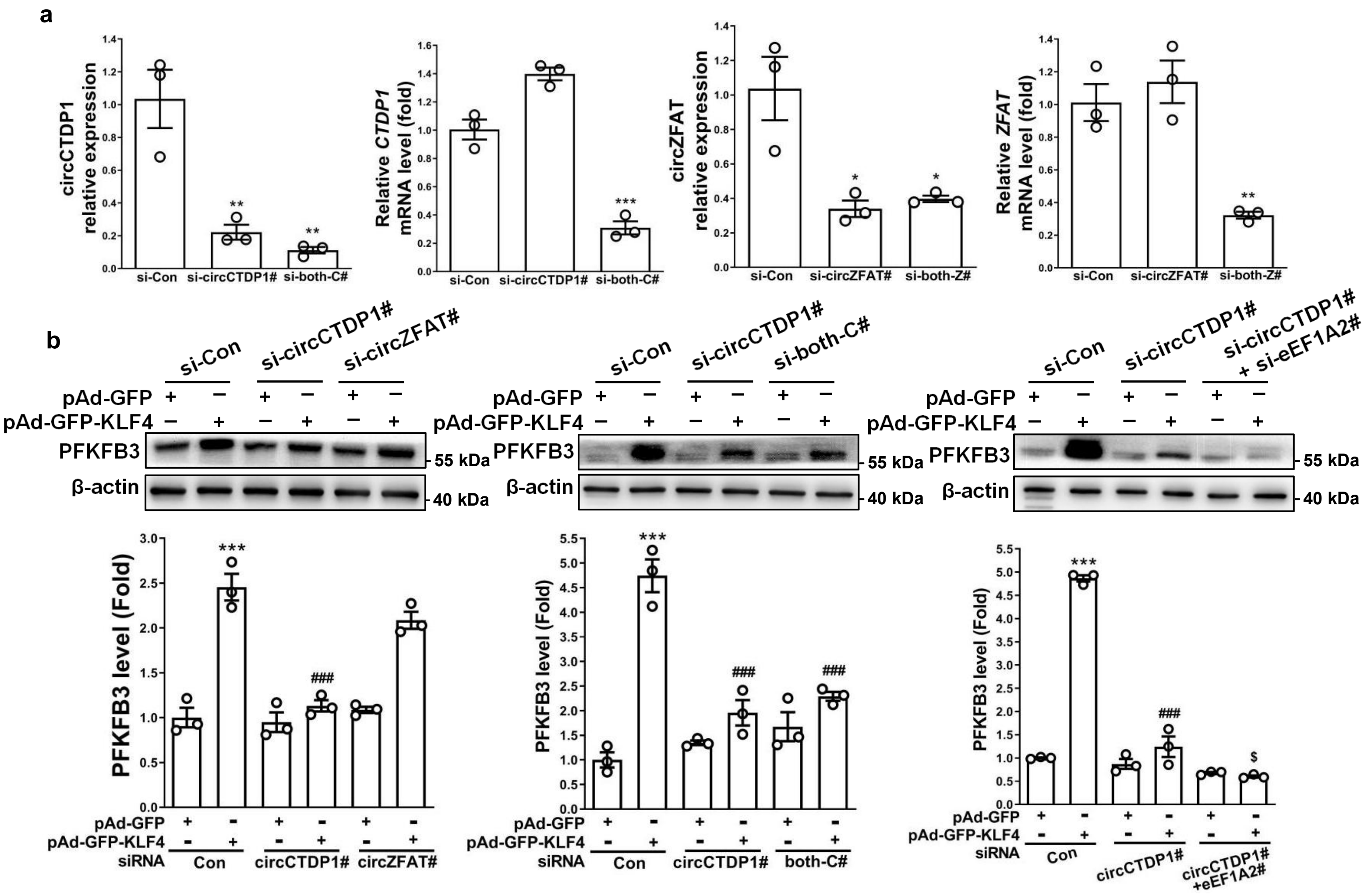

Supplementary Fig. 8 KLF4 upregulates PFKFB3 expression via eEF1A2 and circCTDP1. a qRT-PCR detected the indicated circRNAs and mRNAs in VSMCs transfected with the indicated siRNAs. **P*<0.05, ***P*<0.01 and ****P*<0.005 vs si-Con (n=3 independent experiments, error bars show SEM). **b** VSMCs were transfected with the indicated constructs. PFKFB3 protein levels were measured by immunoblotting and quantified by normalizing to β-actin. ****P*<0.005 vs si-Con+pAd-GFP, ###*P*<0.005 vs si-Con+pAd-GFP-KLF4, $*P*<0.05 vs si-circCTDP1#+pAd-GFP-KLF4 (n=3 independent experiments, error bars show SEM). Unpaired Student's t-tests were performed for **a**. One-way ANOVA with Tukey's multiple comparison tests were performed for **b**.

Supplementary Figure 9

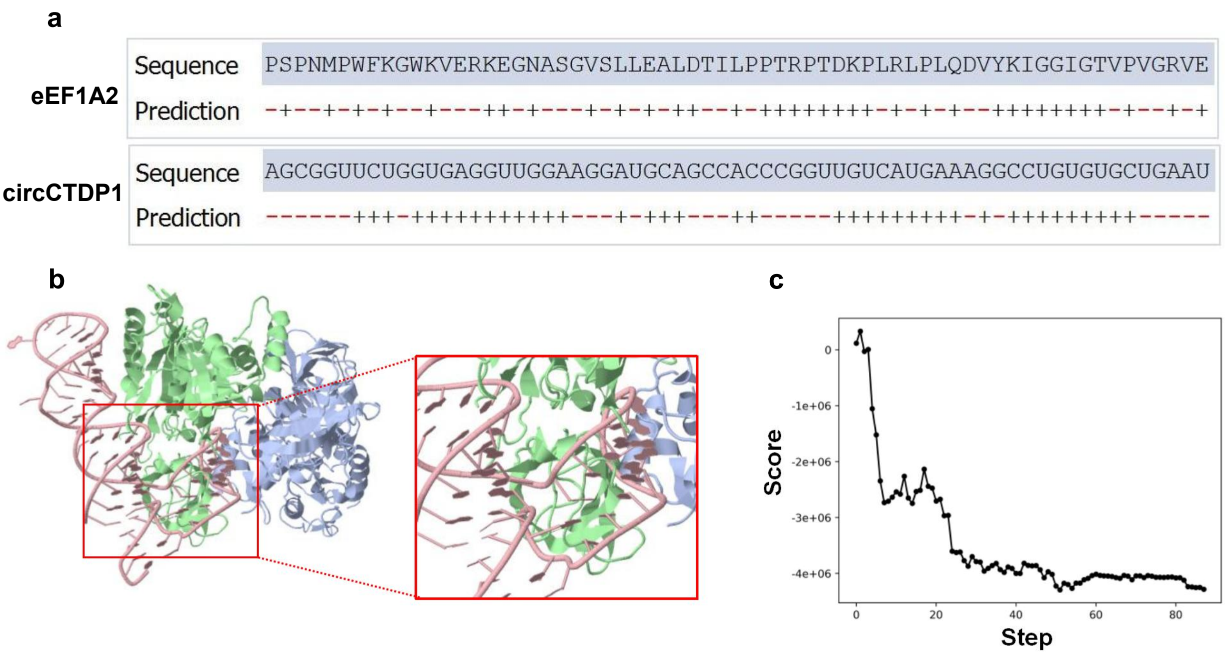

Supplementary Fig. 9 eEF1A2 interacts with circCTDP1. a Prediction of probable binding sites in eEF1A2 and circCTDP1 was carried out by submitting their sequences to PRIdictor servers. Plus signs indicate the predicted binding sites. **b** Graphical representation of three-dimensional structures of the docking model of circCTDP1 with eEF1A2, and magnification of the boxed area showing the details of the binding interface done by NPDock. **c** Refinement of the best docked circCTDP1-eEF1A2 model showing MC score vs. steps of simulation.

Supplementary Figure 10

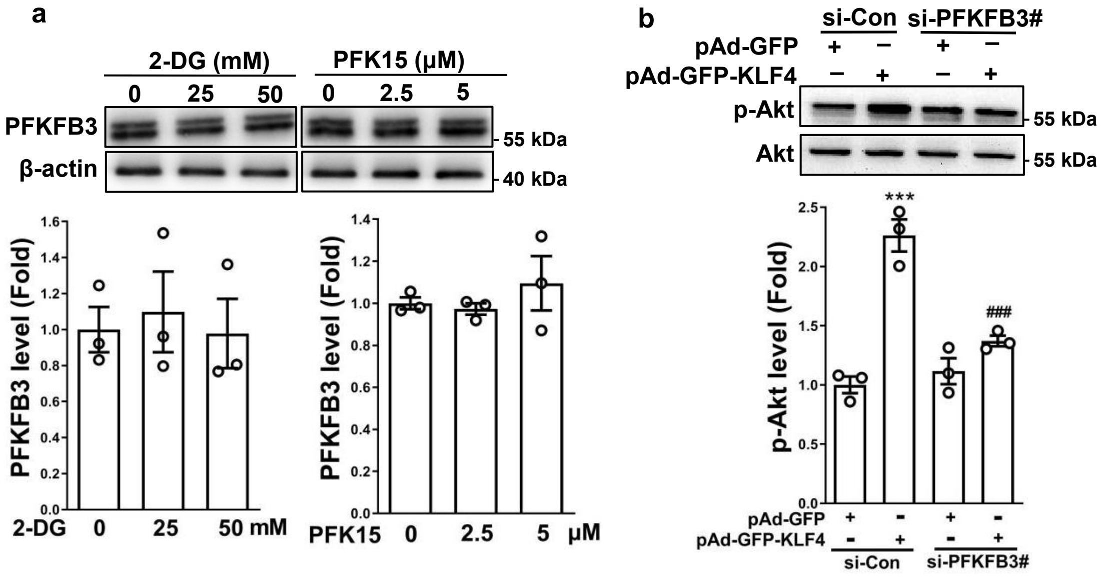

Supplementary Fig. 10 PFKFB3 knockdown decreases KLF4-induced CD123 expression. a VSMCs were treated with the indicated doses of 2-DG or PFK15. PFKFB3 expression levels were measured by immunoblotting and quantified by normalizing to β-actin. n=3 independent experiments, error bars show SEM. One-way ANOVA with Tukey's multiple comparison tests were performed. **b** VSMCs were transfected with the indicated constructs. p-Akt protein levels were measured by immunoblotting and quantified by normalizing to Akt. ****P*<0.005 vs si-Con+pAd-GFP, ###*P*<0.005 vs si-Con+pAd-GFP-KLF4 (n=3 independent experiments, error bars show SEM). One-way ANOVA with Tukey's multiple comparison tests were performed.

Supplementary Figure 11

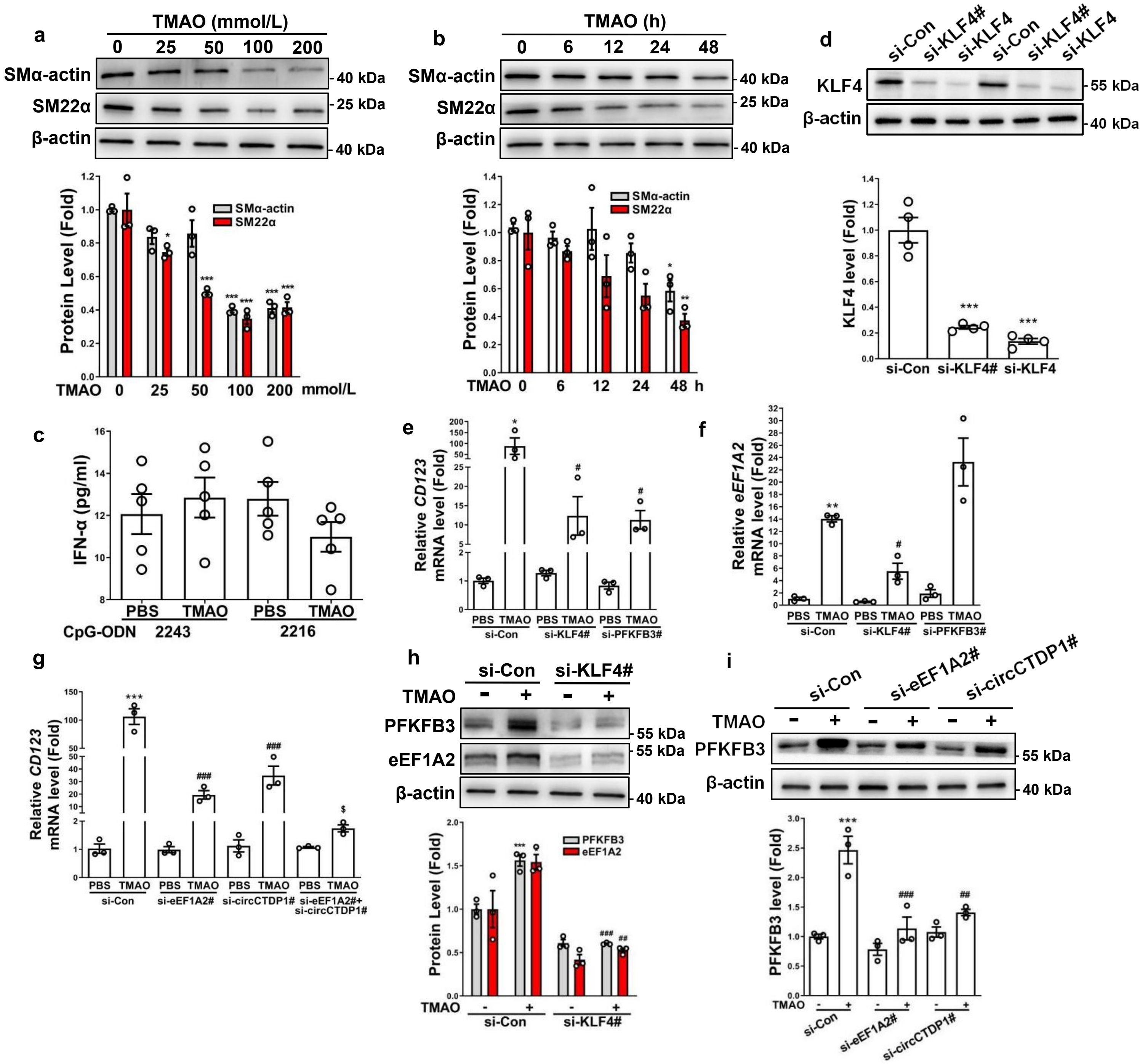

Supplementary Fig. 11 TMAO induces the phenotypic switching of VSMCs to a dysfunctional pDC-like cell by upregulating KLF4 expression. a, b VSMCs were cultured in serum-free medium for 24 h, followed by treatment with the indicated doses of TMAO for the indicated times. SM22α and SMα-actin protein levels were measured by immunoblotting and quantified by normalizing to β-actin. **P*<0.05, ***P*<0.01, and ****P*<0.005 vs TMAO-untreated group (n=3 independent experiments, error bars show SEM). **c** VSMCs were incubated with 200 mM TMAO for 24 h, and then treated with the adjuvant CpG ODN (6 µg/ml) for another 24 h. The culture medium was then collected and IFN-α was detected by ELISA. n=5 independent experiments, error bars show SEM. **d** VSMCs were transfected with two siRNAs targeting KLF4, si-KLF4 and si-KLF4#, for 24 h. KLF4 protein levels were measured by immunoblotting and quantified by normalizing to β-actin. ****P*<0.005 vs si-Con (n=4 independent experiments, error bars show SEM). **e-i** VSMCs were treated as indicated. Expression levels for indicated mRNAs were assessed by qRT-PCR (**e-g**). Expression levels for indicated proteins were measured by immunoblotting and quantified by normalizing to β-actin (**h**, **i**). **P*<0.05, ***P*<0.01, and ****P*<0.005 vs si-Con+PBS group, $^{\#}P$<0.05, $^{\#\#}P$<0.01, and $^{\#\#\#}P$<0.005 vs si-Con+TMAO group, $^{\$}P$<0.05 vs si-circCTDP1+TMAO (n=3 independent experiments, error bars show SEM). One-way ANOVA with Tukey's multiple comparison tests were performed for **a-c** and **e-i**. Unpaired Student's t-tests were performed for **d**.

Supplementary Figure 12

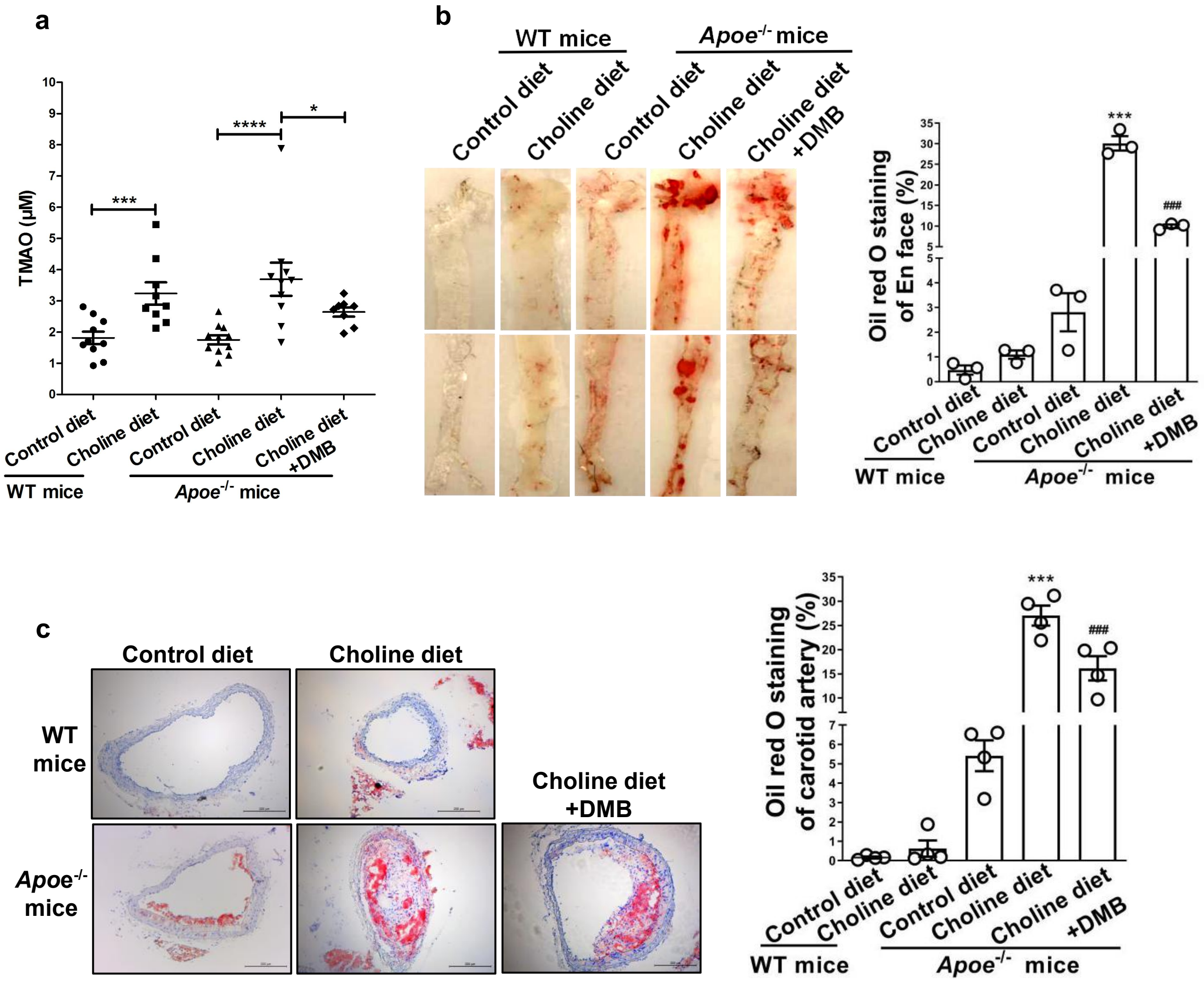

d

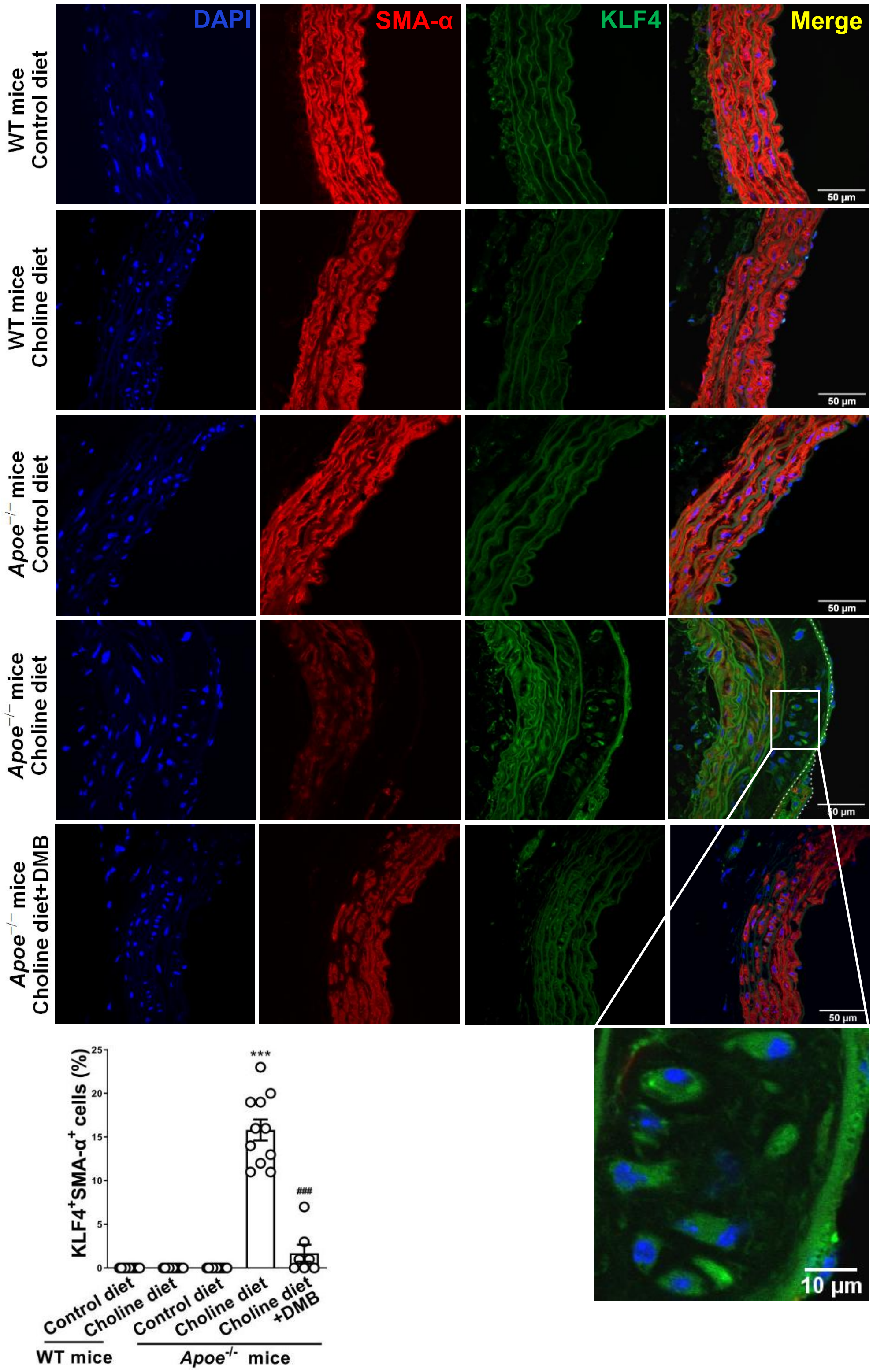

DAPI
SMA-α
KLF4
Merge
WT mice Control diet
WT mice Choline diet
Apoe−/− mice Control diet
Apoe−/− mice Choline diet
Apoe−/− mice Choline diet+DMB
50 μm
10 μm
KLF4+SMA-α+ cells (%)
***
###
Control diet
Choline diet
Control diet
Choline diet
Choline diet +DMB
WT mice
Apoe-/- mice

e

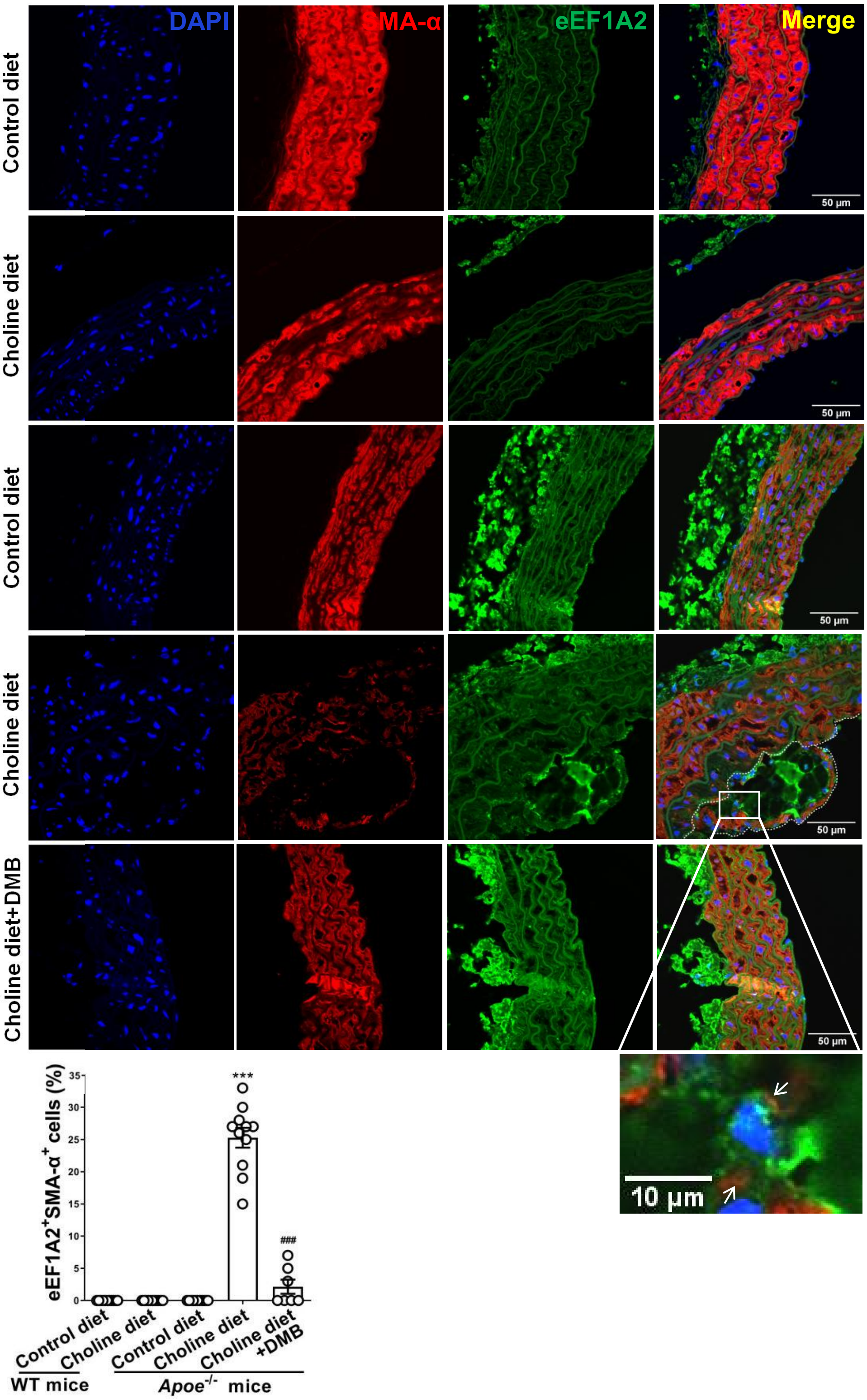
DAPI
SMA-α
eEF1A2
Merge
WT mice
Control diet
WT mice
Choline diet
Apoe−/− mice
Control diet
Apoe−/− mice
Choline diet
Apoe−/− mice
Choline diet+DMB
50 μm
10 μm
eEF1A2+SMA-α+ cells (%)
***
###
Control diet
Choline diet
Control diet
Choline diet
Choline diet +DMB
WT mice
Apoe-/- mice

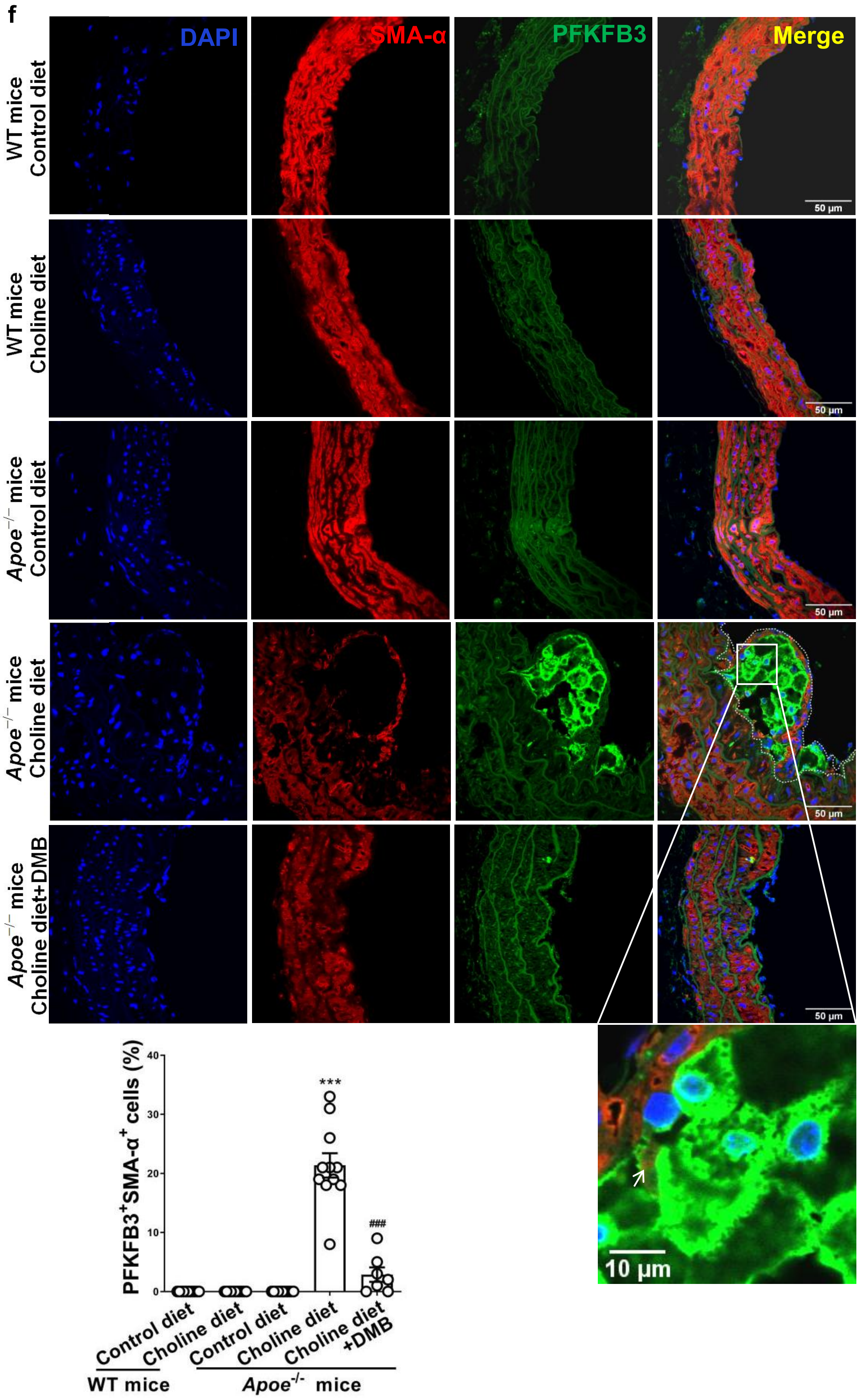
f
DAPI
SMA-α
PFKFB3
Merge
WT mice
Control diet
WT mice
Choline diet
Apoe−/− mice
Control diet
Apoe−/− mice
Choline diet
Apoe−/− mice
Choline diet+DMB
50 μm
10 μm
PFKFB3+SMA-α+ cells (%)
40
30
20
10
0
***
###
Control diet
Choline diet
Control diet
Choline diet
Choline diet +DMB
WT mice
Apoe-/- mice

g

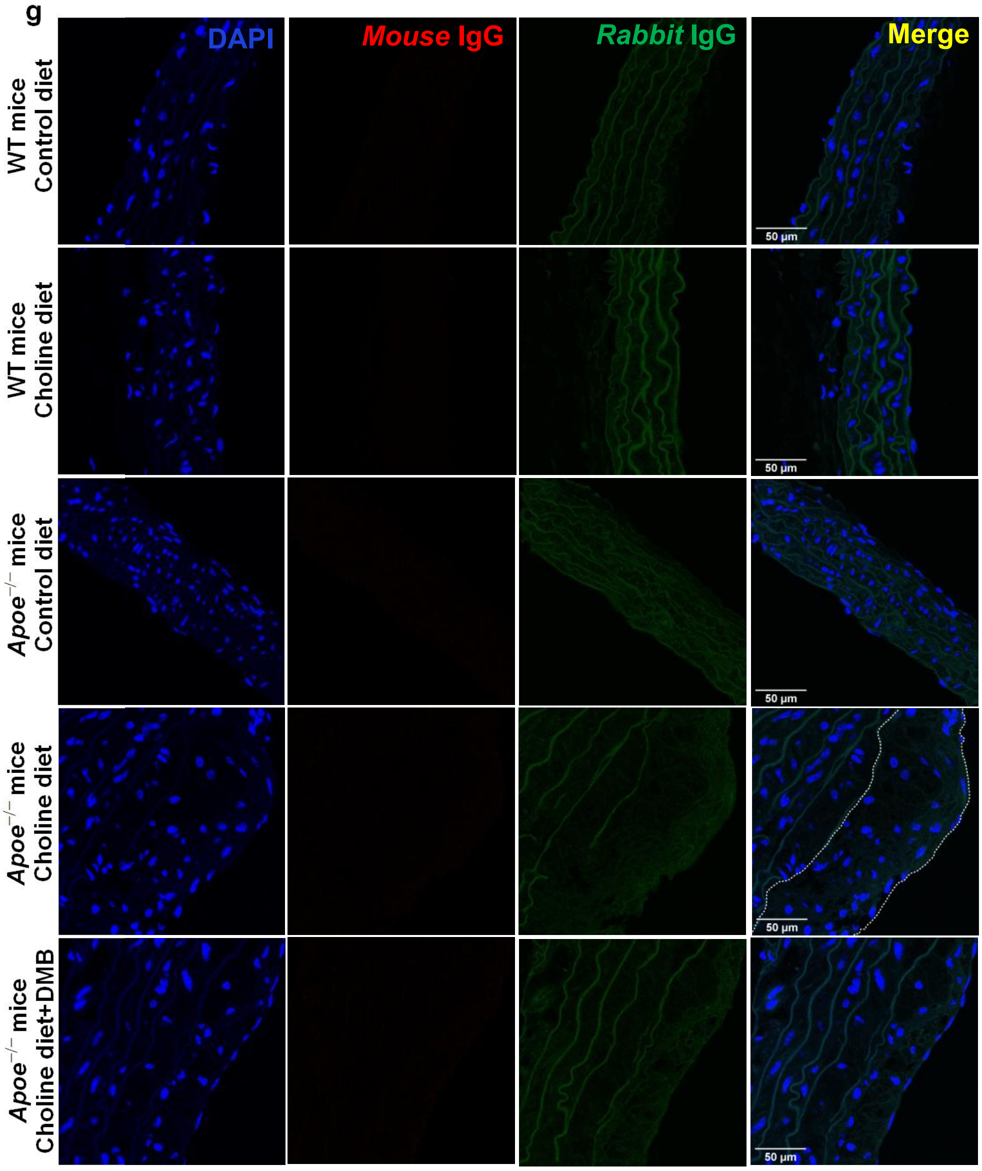

DAPI
Mouse IgG
Rabbit IgG
Merge
WT mice
Control diet
WT mice
Choline diet
Apoe−/− mice
Control diet
Apoe−/− mice
Choline diet
Apoe−/− mice
Choline diet+DMB
50 μm
50 μm
50 μm
50 μm
50 μm

h

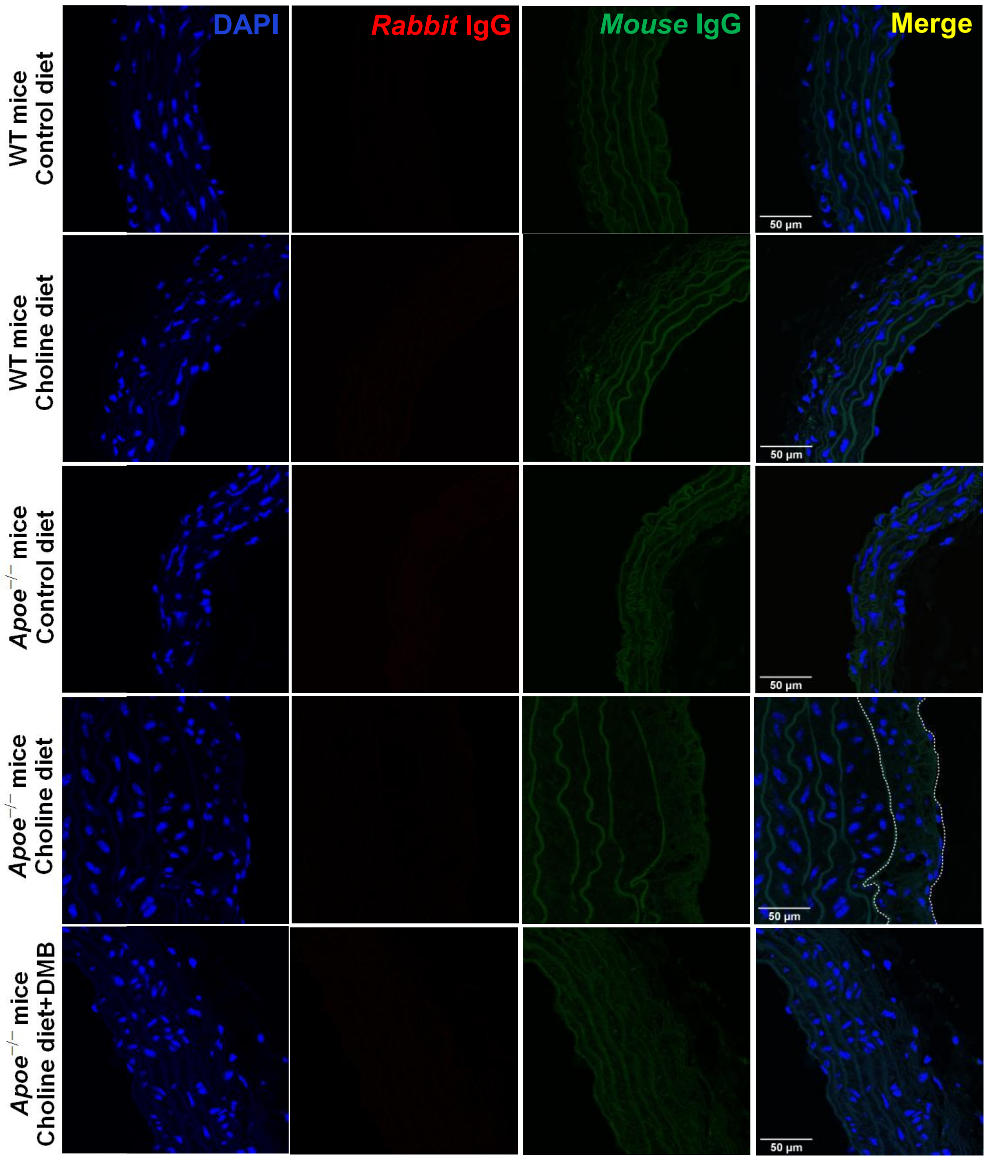
DAPI
Rabbit IgG
Mouse IgG
Merge
WT mice
Control diet
WT mice
Choline diet
Apoe−/− mice
Control diet
Apoe−/− mice
Choline diet
Apoe−/− mice
Choline diet+DMB
50 μm

Supplementary Fig. 12 TMAO enhances atherosclerosis by inducing VSMC phenotypic switching in Apoe$^{-/-}$ mice. a-h WT or Apoe$^{-/-}$ mice were fed with either a normal diet (contains 0.07-0.08% total choline, wt/wt) or a normal diet supplemented with high amounts of additional choline (1.3%) in the presence *versus* absence of DMB (1.0%, v/v, provided in the drinking water) for 14 weeks. The circulating TMAO levels were quantified using LC/MS/MS (**a**). *P<0.05 vs choline diet, ***P<0.005 and ****P<0.001 vs control diet (n=8-11 independent samples, error bars show SEM). Representative Oil Red O staining and en face analysis of atherosclerotic lesions in the whole aorta (**b**). ***P<0.005 vs control diet Apoe$^{-/-}$ mice, $^{###}P$<0.005 vs choline diet Apoe$^{-/-}$ mice (n=3 independent experiments, error bars show SEM). Oil red O staining and cross sectional analysis of atherosclerotic lesions in the carotid arteries (bars=200 μm) (**c**). ***P<0.005 vs control diet Apoe$^{-/-}$ mice, $^{###}P$<0.005 vs choline diet Apoe$^{-/-}$ mice (n=4 independent experiments, error bars show SEM). Representative immunofluorescent staining of SMA-α (red), KLF4 (green) and DAPI (blue) on cross sections of the mouse aortic root (bars=50 μm) and quantification of the frequency of KLF4^{+}SMA-α^{+} cells as a percent of total DAPI^{+} cells in the core region of atherosclerotic lesions (**d**). ***P<0.005 vs control diet Apoe$^{-/-}$ mice, $^{###}P$<0.005 vs choline diet Apoe$^{-/-}$ mice (n=7 or 11 independent experiments, error bars show SEM). Representative immunofluorescent staining of SMA-α (red), eEF1A2 (green) and DAPI (blue) on cross sections of the mouse aortic root (bars=50 μm) and quantification of the frequency of eEF1A2^{+}SMA-α^{+} cells as a percent of total DAPI^{+} cells in the core region of atherosclerotic lesions (**e**). ***P<0.005 vs control diet Apoe$^{-/-}$ mice, $^{###}P$<0.005 vs choline diet Apoe$^{-/-}$ mice (n=7 or 11 independent experiments, error bars show SEM). Representative immunofluorescent staining of SMA-α (red), PFKFB3 (green) and DAPI (blue) on cross sections of the mouse aortic root (bars=50 μm) and quantification of the frequency of PFKFB3^{+}SMA-α^{+} cells as a percent of total DAPI^{+} cells in the core region of atherosclerotic lesions (**f**). ***P<0.005 vs control diet Apoe$^{-/-}$ mice, $^{###}P$<0.005 vs choline diet Apoe$^{-/-}$ mice (n=7 or 11 independent experiments, error bars show SEM). One-way ANOVA with Tukey's multiple comparison tests were performed. Representative images of immunohistochemical quality controls (**g, h**).

Supplementary Figure 13

Fig. 3

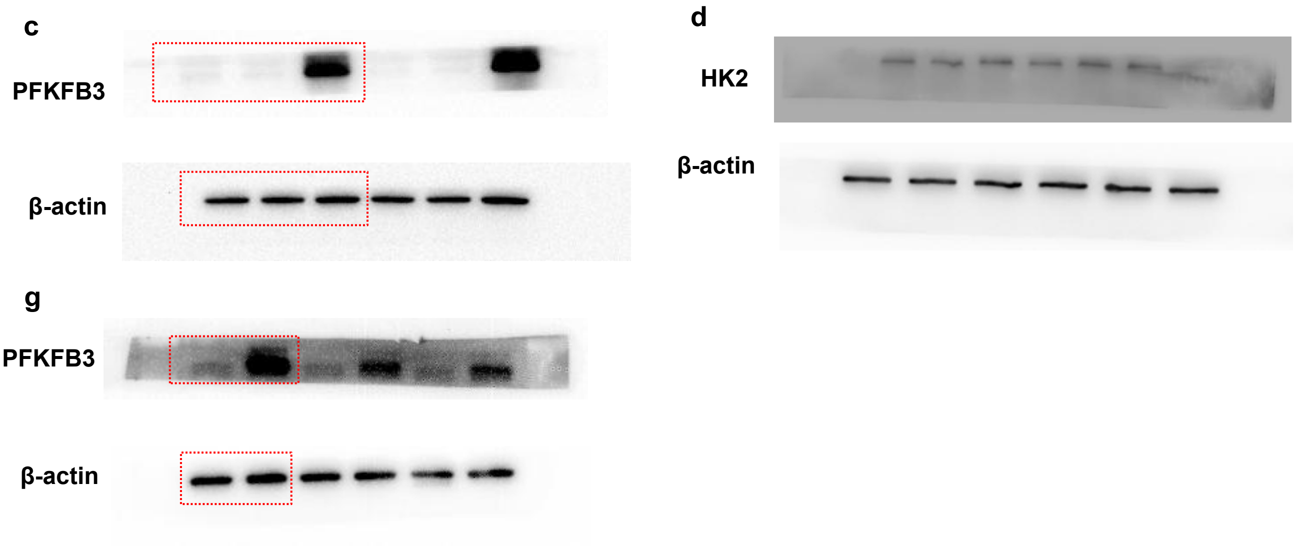

Fig. 4

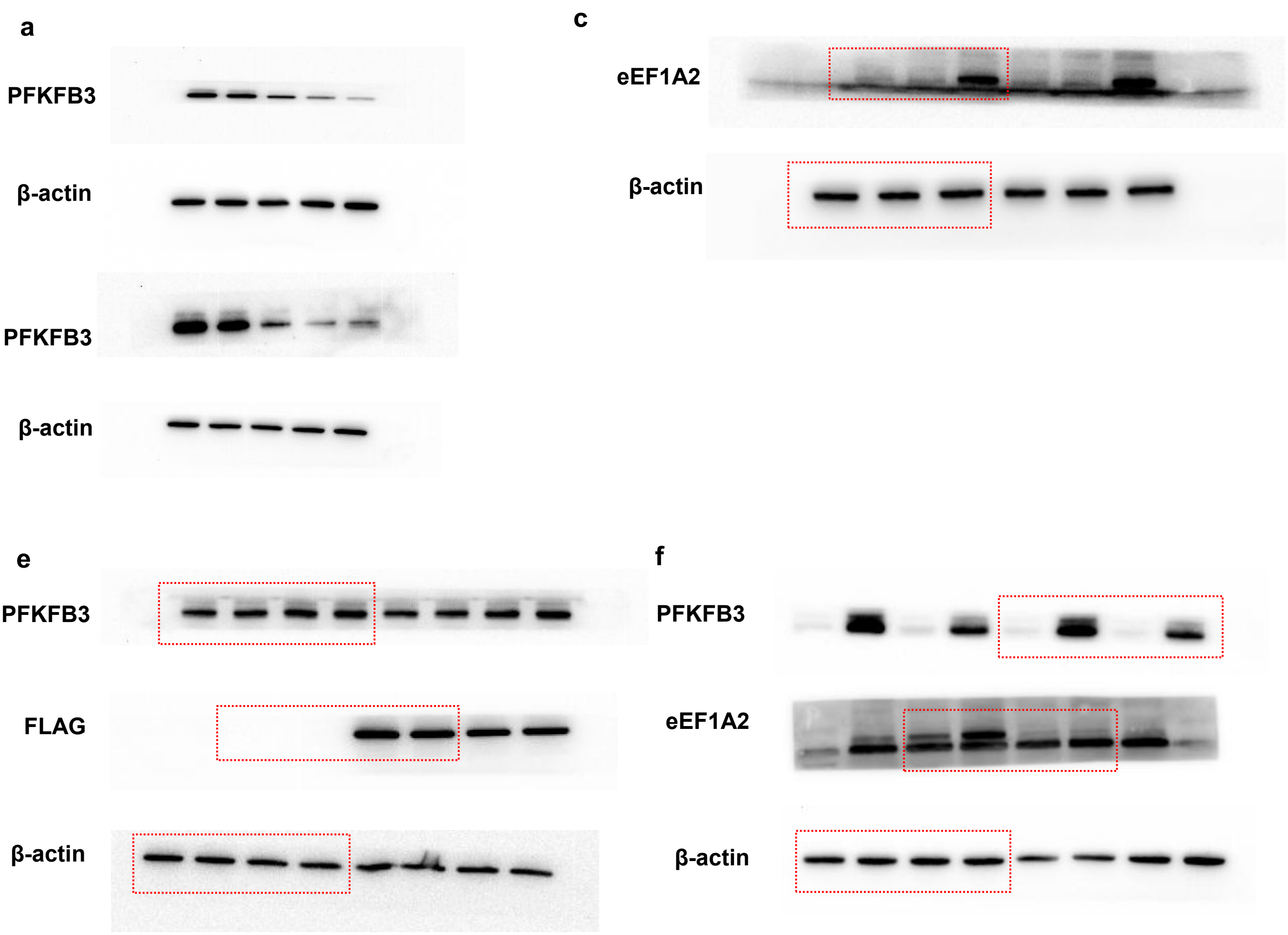

Supplementary Fig. 13 Full-width of original blots for Fig. 3 and 4. Panels indicated in this supplemental figure correspond to those in the main article. Red rectangles indicate the area shown in each figure pane.

Supplementary Figure 14

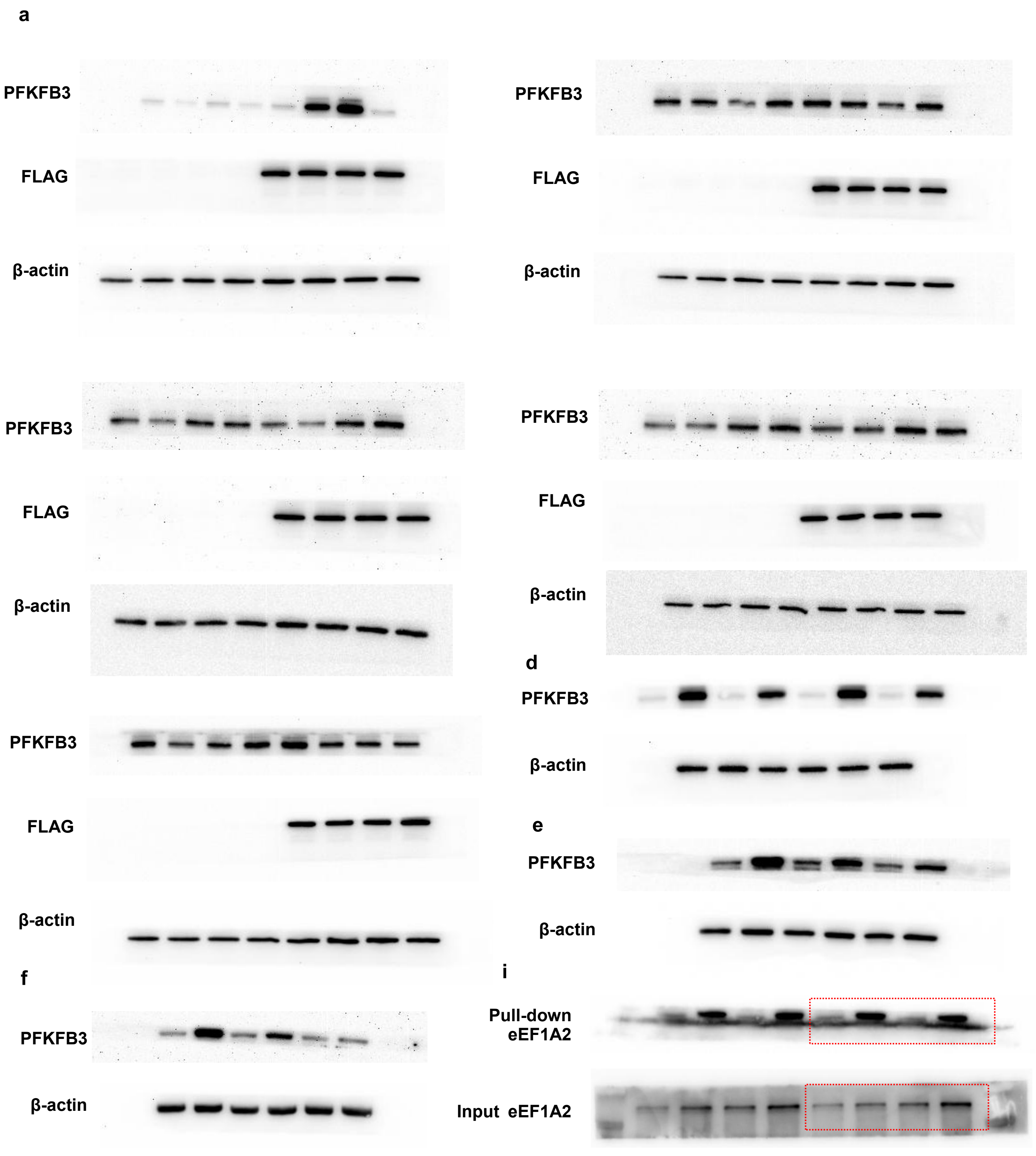

Supplementary Fig. 14 Full-width of original blots for Fig. 5. Panels indicated in this supplemental figure correspond to those in the main article. Red rectangles indicate the area shown in each figure pane.

Supplementary Figure 15

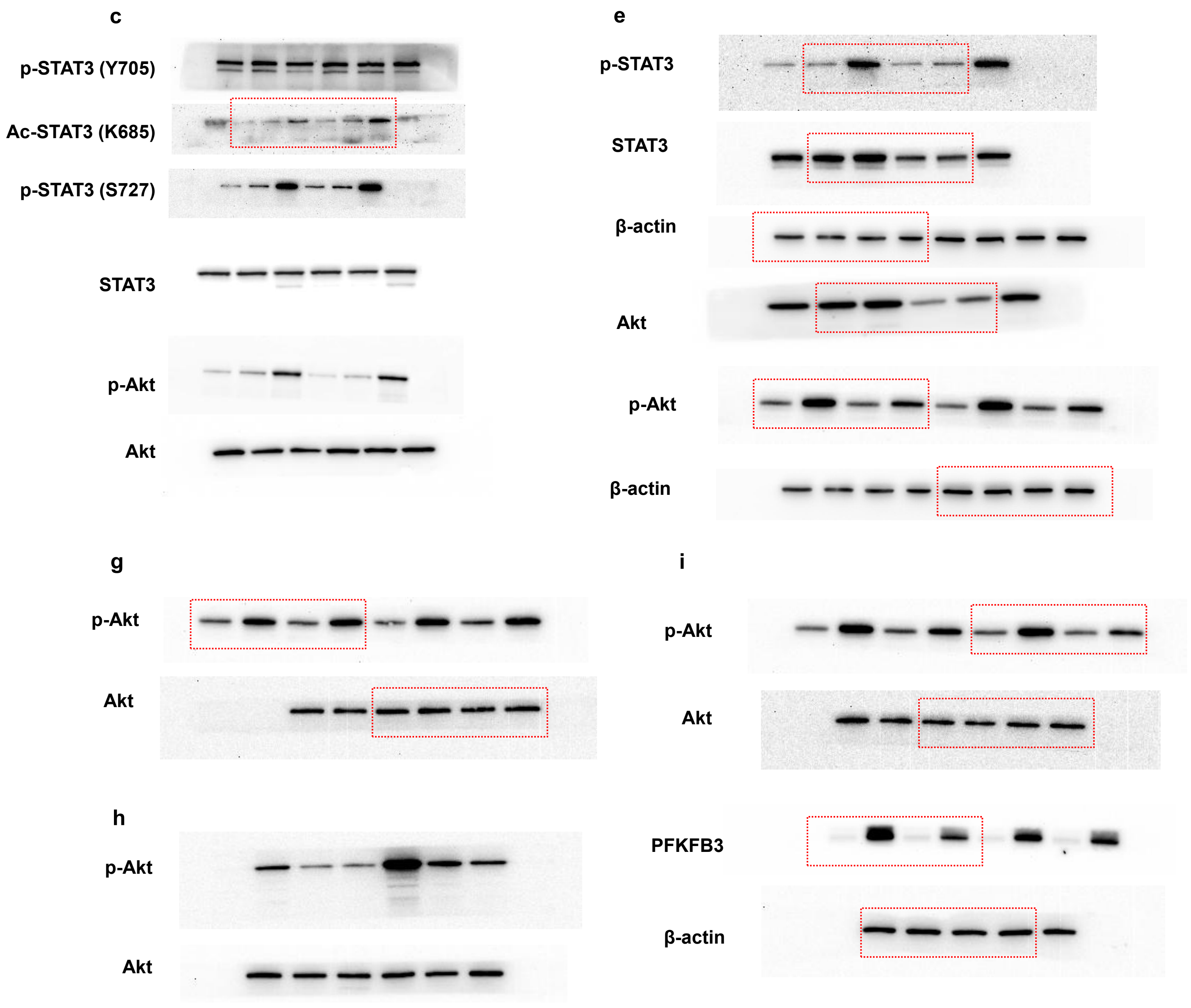

Supplementary Fig. 15 Full-width of original blots for Fig. 6. Panels indicated in this supplemental figure correspond to those in the main article. Red rectangles indicate the area shown in each figure pane.

Supplementary Figure 16

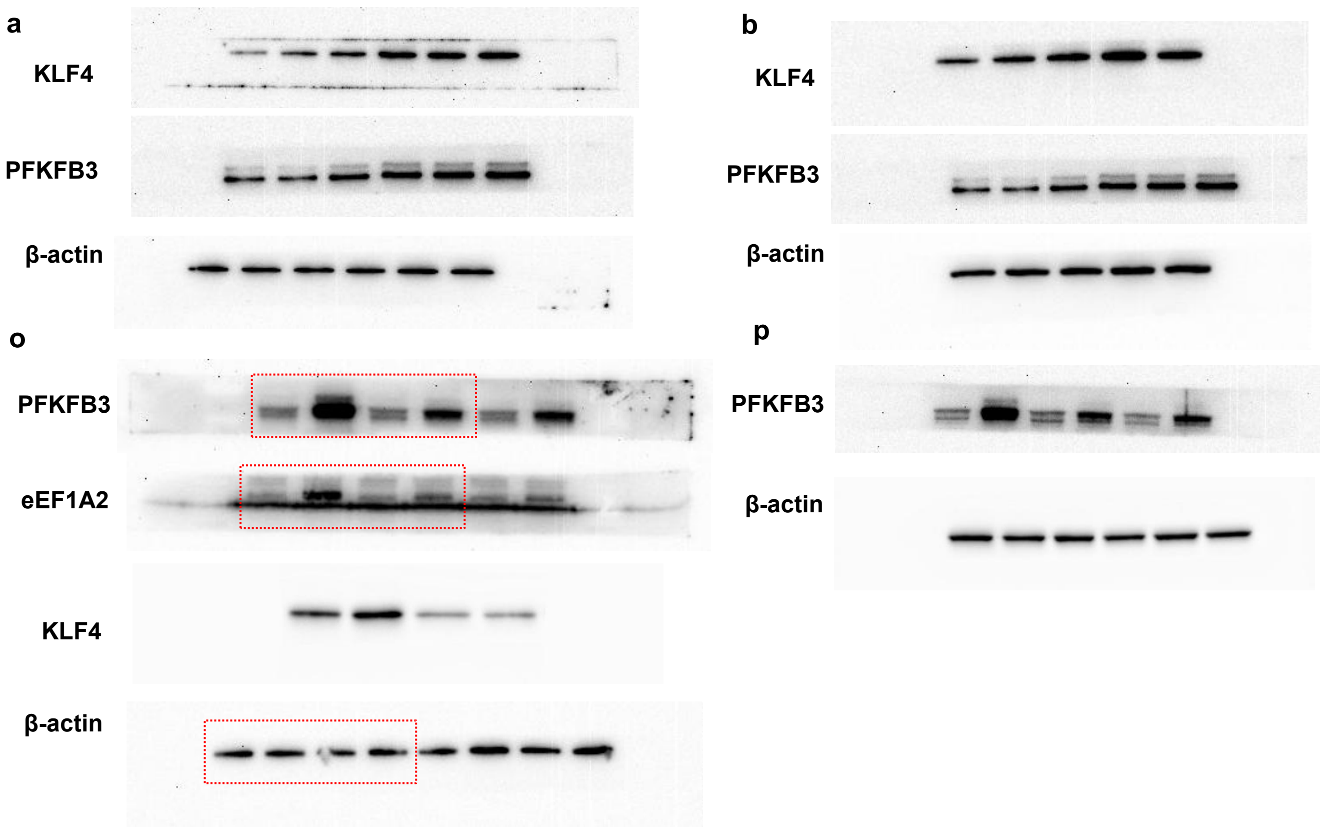

Supplementary Fig. 16 Full-width of original blots for Fig. 7. Panels indicated in this supplemental figure correspond to those in the main article. Red rectangles indicate the area shown in each figure pane.

Supplementary Figure 17

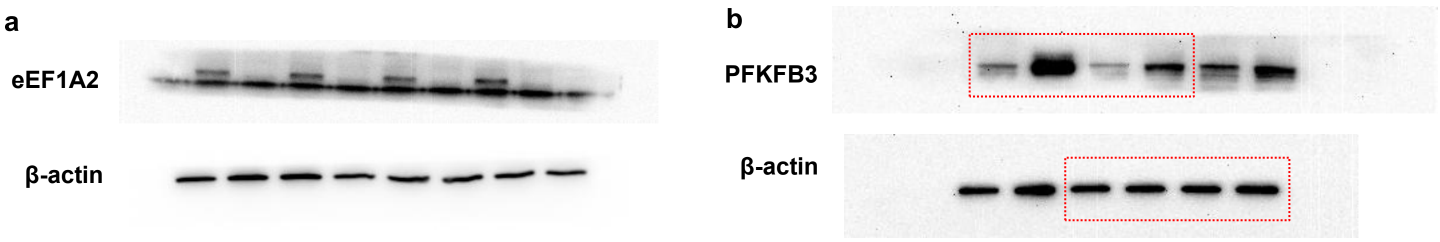

Supplementary Fig. 17 Full-width of original blots for Supplementary Fig. 4. Panels indicated in this supplemental figure correspond to those in Supplementary Fig. 4. Red rectangles indicate the area shown in each figure pane.

Supplementary Figure 18

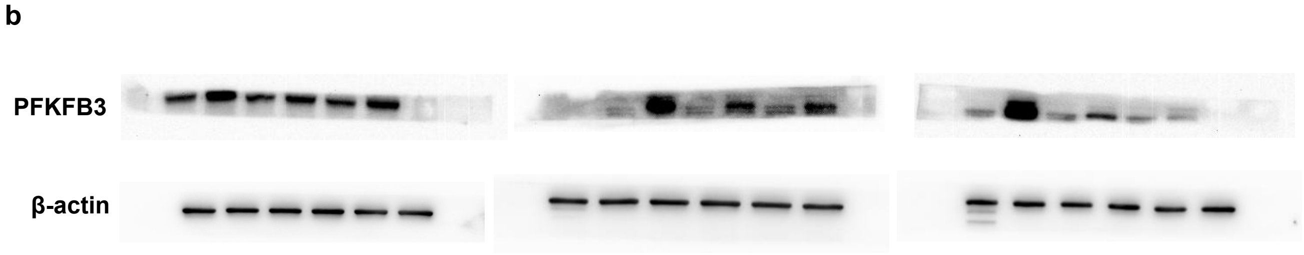

Supplementary Fig. 18 Full-width of original blots for Supplementary Fig. 8. Panels indicated in this supplemental figure correspond to those in Supplementary Fig. 8.

Supplementary Figure 19

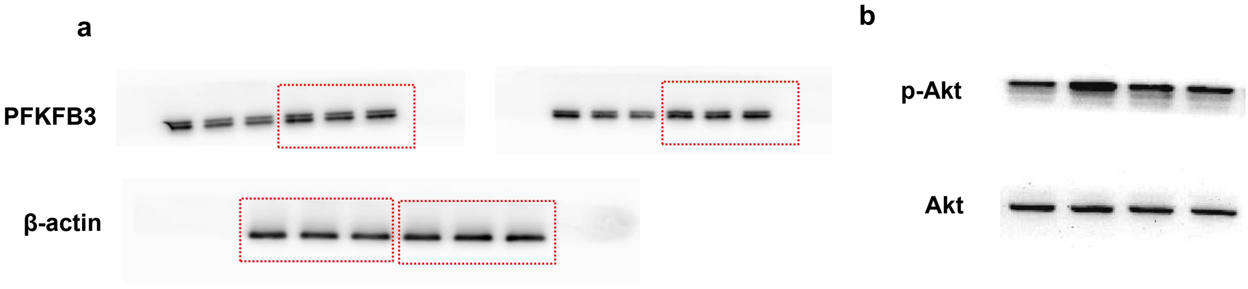

Supplementary Fig. 19 Full-width of original blots for Supplementary Fig. 10. Panels indicated in this supplemental figure correspond to those in Supplementary Fig. 10. Red rectangles indicate the area shown in each figure pane.

Supplementary Figure 20

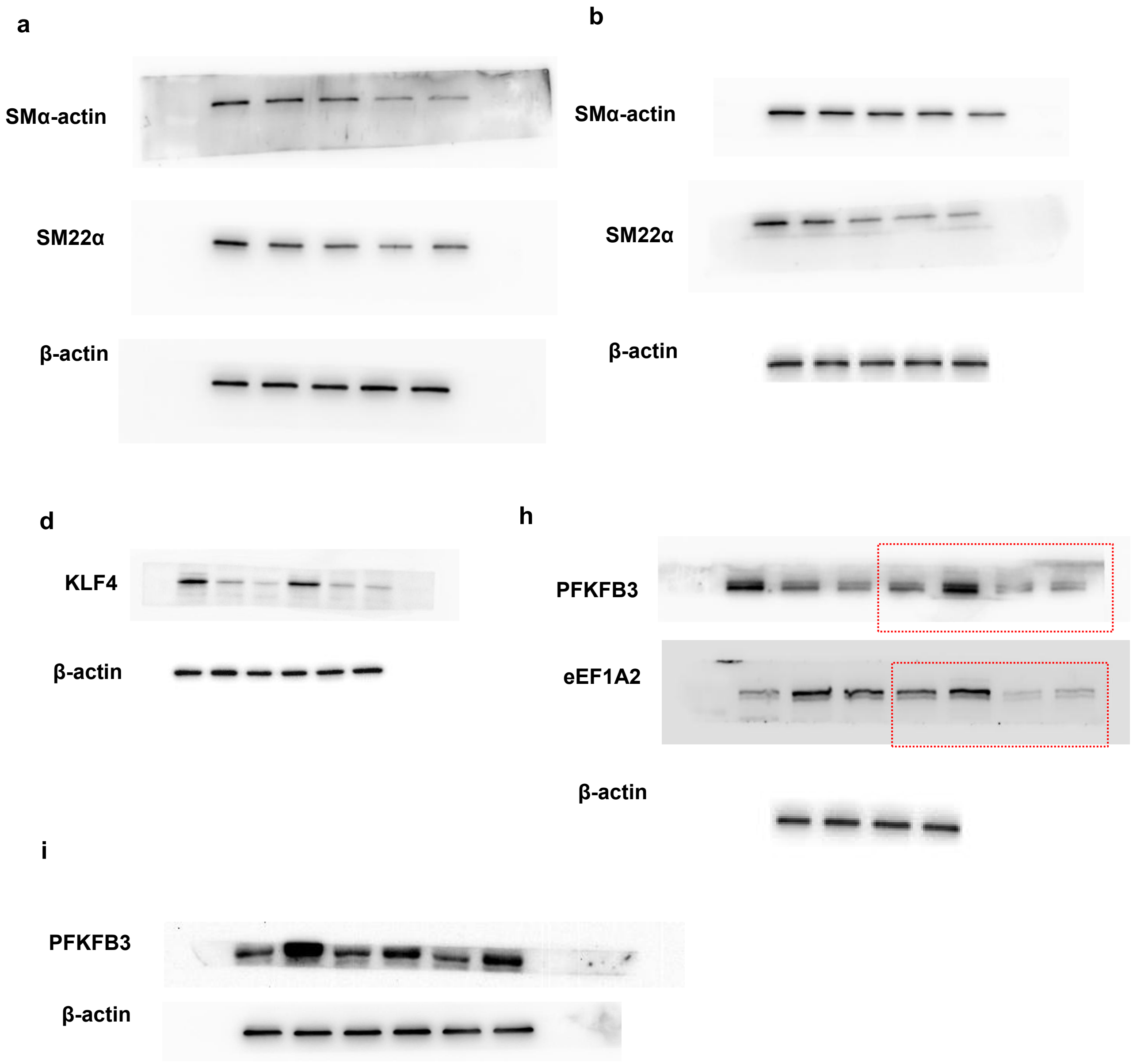

Supplementary Fig. 20 Full-width of original blots for Supplementary Fig. 11. Panels indicated in this supplemental figure correspond to those in Supplementary Fig. 11. Red rectangles indicate the area shown in each figure pane.
