# Supplementary material for: KLF4-PFKFB3-driven glycolysis is essential for phenotypic switching of vascular smooth muscle cells
Source: Commun Biol. 2022 Dec 5;5:1332. doi: 10.1038/s42003-022-04302-y (PMC9722670; doi:10.1038/s42003-022-04302-y)
Supplement: Supplementary file 3 — Description of Additional Supplementary Data [file 42003_2022_4302_MOESM3_ESM.docx]

**Description of Additional Supplementary Files**

**File name:** Supplementary Data

**Description:** The source data behind the graphs in the paper.
