# Supplementary material for: KLF4-PFKFB3-driven glycolysis is essential for phenotypic switching of vascular smooth muscle cells
Source: Commun Biol. 2022 Dec 5;5:1332. doi: 10.1038/s42003-022-04302-y (PMC9722670; doi:10.1038/s42003-022-04302-y)
Supplement: Supplementary file 5 — Reporting Summary [file 42003_2022_4302_MOESM5_ESM.pdf]

nature portfolio

Corresponding author(s): Xinhua Zhang

Last updated by author(s): Oct 28, 2022

Reporting Summary

Nature Portfolio wishes to improve the reproducibility of the work that we publish. This form provides structure for consistency and transparency in reporting. For further information on Nature Portfolio policies, see our Editorial Policies and the Editorial Policy Checklist.

Statistics

For all statistical analyses, confirm that the following items are present in the figure legend, table legend, main text, or Methods section.

n/a	Confirmed	
☐	☒	The exact sample size (*n*) for each experimental group/condition, given as a discrete number and unit of measurement
☐	☒	A statement on whether measurements were taken from distinct samples or whether the same sample was measured repeatedly
☐	☒	The statistical test(s) used AND whether they are one- or two-sided<br>*Only common tests should be described solely by name; describe more complex techniques in the Methods section.*
☒	☐	A description of all covariates tested
☒	☐	A description of any assumptions or corrections, such as tests of normality and adjustment for multiple comparisons
☐	☒	A full description of the statistical parameters including central tendency (e.g. means) or other basic estimates (e.g. regression coefficient) AND variation (e.g. standard deviation) or associated estimates of uncertainty (e.g. confidence intervals)
☒	☐	For null hypothesis testing, the test statistic (e.g. *F*, *t*, *r*) with confidence intervals, effect sizes, degrees of freedom and *P* value noted<br>*Give P values as exact values whenever suitable.*
☒	☐	For Bayesian analysis, information on the choice of priors and Markov chain Monte Carlo settings
☒	☐	For hierarchical and complex designs, identification of the appropriate level for tests and full reporting of outcomes
☒	☐	Estimates of effect sizes (e.g. Cohen's *d*, Pearson's *r*), indicating how they were calculated

Our web collection on statistics for biologists contains articles on many of the points above.

Software and code

Policy information about availability of computer code

Data collection	-ABI 7500 FAST system (Life Technologies) was used for RT-qPCR data acquisition.<br>-EvolutionCapt FX6 software was used for chemiluminescence detection of Western blots.<br>-LAS V4.12 software was used for imaging of HE staining.<br>-LAS-AF-Lite_2.5.2_6939 software was used for confocal microscope.<br>-Analyst v1.5.2 software (Applied Biosystems) was used for PEP and TCA cycle intermediate measurements.<br>-FAC Sverse (FC500 MPL Beckman) system was used for the Flow cytometry detection.<br>-Seahorse Bioscience XF-24 Extracellular Flux Analyzer system was used for the extracellular acidification rate measurement.
Data analysis	-GraphPad Prism 9 software was used for statistical analysis.<br>-Image J/FIJI software was used for processing and quantifing images.<br>-GeneSpring software V12.1 (Agilent Technologies) was used for the mRNA microarray analysis.<br>-Nano-HPLC (EASY-nLC1200) coupled to Q-Exactive mass spectrometry (Thermo Finnigan) was used for the TMT-based LC-MS/MS analysis.<br>-MeV_4_9_0 software was used for drawing the heat map.

For manuscripts utilizing custom algorithms or software that are central to the research but not yet described in published literature, software must be made available to editors and reviewers. We strongly encourage code deposition in a community repository (e.g. GitHub). See the Nature Portfolio guidelines for submitting code & software for further information.

Data

Policy information about availability of data

All manuscripts must include a data availability statement. This statement should provide the following information, where applicable:

- Accession codes, unique identifiers, or web links for publicly available datasets
- A description of any restrictions on data availability
- For clinical datasets or third party data, please ensure that the statement adheres to our policy

All datasets generated and/or analyzed during this study are available from the corresponding author on reasonable request.

Human research participants

Policy information about studies involving human research participants and Sex and Gender in Research.

Reporting on sex and gender	ALL the tissues were from the male.
Population characteristics	Human vascular samples were obtained from thirteen patients, seven with hypertension and six without hypertension. Patients who had hypertension at least 10 years managed blood pressure by using hypotensor.
Recruitment	The renal carcinoma patients with or withour hypertension were undergoing surgery.
Ethics oversight	The protocols for human studies were approved by the ethics committee of the Second Hospital of Hebei Medical University. Each of the surgical patients gave informed consent before donating tissue.

Note that full information on the approval of the study protocol must also be provided in the manuscript.

Field-specific reporting

Please select the one below that is the best fit for your research. If you are not sure, read the appropriate sections before making your selection.

☒ Life sciences ☐ Behavioural & social sciences ☐ Ecological, evolutionary & environmental sciences

For a reference copy of the document with all sections, see nature.com/documents/nr-reporting-summary-flat.pdf

Life sciences study design

All studies must disclose on these points even when the disclosure is negative.

Sample size	The sample size was determined as sufficient for statistical analysis.
Data exclusions	No data were excluded from the analysis.
Replication	All attempts for replication were successful in this study.
Randomization	Randomization was not applied.
Blinding	Our experiments were not blinded, because we have controls run side-by-side with the experimental samples.

Reporting for specific materials, systems and methods

We require information from authors about some types of materials, experimental systems and methods used in many studies. Here, indicate whether each material, system or method listed is relevant to your study. If you are not sure if a list item applies to your research, read the appropriate section before selecting a response.

Materials & experimental systems

n/a	Involved in the study	
☐	☒	Antibodies
☐	☒	Eukaryotic cell lines
☒	☐	Palaeontology and archaeology
☐	☒	Animals and other organisms
☒	☐	Clinical data
☒	☐	Dual use research of concern

Methods

n/a	Involved in the study	
☒	☐	ChIP-seq
☐	☒	Flow cytometry
☒	☐	MRI-based neuroimaging

Antibodies

Antibodies used

Antibodies used for Western blot analysis:
anti-PFKFB3 (1:1000, ab181861), anti-HK2 (1:1000, ab209847), anti-eEF1A2 (1:1000, 16091-1-AP), anti-KLF4 (1:1000, 11880-1-AP), anti-SM α-actin (1:1000, ab32575), anti-SM22α (1:1000, ab14106), anti-phospho-Stat3 (Tyr 705) (1:1000, Cell Signaling, catalog no. 9145), anti-phospho-Stat3 (Ser 727) (1:1000, Cell Signaling, catalog no. 94994), anti-acetyl-Stat3 (Lys 685) (1:1000, Cell Signaling, catalog no. 2523), anti-Stat3 (1:1000, Cell Signaling, catalog no. 9139), anti-phospho-Akt (Ser/Thr) (1:1000, Cell Signaling, catalog no. 9611), anti-Akt (1:1000, Cell Signaling, catalog no. 4691), anti-FLAG (1:2000, Sigma, catalog no. F365), anti-β-actin (1:2000, sc-47778), and HRP-conjugated secondary antibody (1:5000, Rockland).

Antibodies used for ISH-PLA staining:
anti-SM α-actin (ab32575 or ab240654, Abcam), anti-CD123 (ab21562, Abcam), anti-LY6D (HPA024755, Merck), anti-H3K4dime (ab6000, Abcam), and anti-Biotin (ab53494, Abcam).

Antibodies used for the ISH/protein staining:
anti-eEF1A2 (16091-1-AP, Proteintech).

Antibodies used for immunostaining:
anti-CD123 (1:100 dilution, ab21562, Abcam), anti-KLF4 (1:100 dilution, ab215036, Abcam), anti-SM α-actin (ab32575 or ab240654, Abcam), anti-CD68 (ab213363, Abcam), anti-PFKFB3 (ab181861, Abcam), anti-LY6D (HPA024755, Merck), anti-CD11c (ab11029, Abcam), and anti-eEF1A2 (16091-1-AP, Proteintech).

Antibodies used for flow cytometry analysis:
anti-APC-CD123 (306012, BioLegend) and anti-Isotype control antibody (400122, BioLegend).

Validation

All antibodies validation are available on the manufacturer's homepage.

Eukaryotic cell lines

Policy information about cell lines and Sex and Gender in Research

Cell line source(s)	Human aortic smooth muscle cells (HASMCs) were from ScienCell (ScienCell, no. 6110).
Authentication	HASMCs were used in our previous work (Xin-hua Zhang, et al, Hypertension, 2015; 66:412-421).
Mycoplasma contamination	There were no mycoplasma contaminatin.
Commonly misidentified lines (See ICLAC register)	None.

Animals and other research organisms

Policy information about studies involving animals; ARRIVE guidelines recommended for reporting animal research, and Sex and Gender in Research

Laboratory animals	4-week-old male wild-type (WT) C57BL/6J mice or apolipoprotein E-null (Apoe−/−) C57BL/6J mice were used.
Wild animals	No wild animals were used in this study.
Reporting on sex	Male mice were used in this study.
Field-collected samples	None.
Ethics oversight	All animal studies were approved by the Institutional Animal Care and Use Committee of Hebei Medical University (approval ID: HebMU 20080026) and all efforts were made to minimize suffering.

Note that full information on the approval of the study protocol must also be provided in the manuscript.

Flow Cytometry

Plots

Confirm that:

☒ The axis labels state the marker and fluorochrome used (e.g. CD4-FITC).

☒ The axis scales are clearly visible. Include numbers along axes only for bottom left plot of group (a 'group' is an analysis of identical markers).

☒ All plots are contour plots with outliers or pseudocolor plots.

☒ A numerical value for number of cells or percentage (with statistics) is provided.

Methodology

Sample preparation	HASMCs infected with pAd-GFP or pAd-GFP-KLF4 were collected and washed with PBS and then resuspended in 100 µl FACS buffer.
Instrument	FC500 MPL Beckman
Software	FAC Sverse
Cell population abundance	Cells that reached about 90% confluence were used.
Gating strategy	Cells were gated based on FSC/SSC profile.

☒ Tick this box to confirm that a figure exemplifying the gating strategy is provided in the Supplementary Information.
